# Bone marrow stromal cells (BMSCs CD45^‐^/CD44^+^/CD73^+^/CD90^+^) isolated from osteoporotic mice SAM/P6 as a novel model for osteoporosis investigation

**DOI:** 10.1111/jcmm.16667

**Published:** 2021-06-01

**Authors:** Mateusz Sikora, Agnieszka Śmieszek, Krzysztof Marycz

**Affiliations:** ^1^ The Department of Experimental Biology The Faculty of Biology and Animal Science University of Environmental and Life Sciences Wroclaw Wroclaw Poland; ^2^ International Institute of Translational Medicine Malin Poland

**Keywords:** bone marrow, bone marrow stromal cells, cellular model, osteogenic markers, senile osteoporosis, stromal cells

## Abstract

Available therapies aimed at treating age‐related osteoporosis are still insufficient. Therefore, designing reliable in vitro model for the analysis of molecular mechanisms underlying senile osteoporosis is highly required. We have isolated and characterized progenitor cells isolated from bone marrow (BMSCs) of osteoporotic mice strain SAM/P6 (BMSC_SAM/P6_). The cytophysiology of BMSC_SAM/P6_ was for the first time compared with BMSCs isolated from healthy BALB/c mice (BMSC_BALB/c_). Characterization of the cells included evaluation of their multipotency, morphology and determination of specific phenotype. Viability of BMSCs cultures was determined in reference to apoptosis profile, metabolic activity, oxidative stress, mitochondrial membrane potential and caspase activation. Additionally, expression of relevant biomarkers was determined with RT‐qPCR. Obtained results indicated that BMSC_SAM/P6_ and BMSC_BALB/c_ show the typical phenotype of mesenchymal stromal cells (CD44+, CD73+, CD90+) and do not express CD45. Further, BMSC_SAM/P6_ were characterized by deteriorated multipotency, decreased metabolic activity and increased apoptosis occurrence, accompanied by elevated oxidative stress and mitochondria depolarisation. The transcriptome analyses showed that BMSC_SAM/P6_ are distinguished by lowered expression of molecules crucial for proper osteogenesis, including *Coll‐1*, *Opg* and *Opn*. However, the expression of *Trap*, *DANCR1* and miR‐124‐3p was significantly up‐regulated. Obtained results show that BMSC_SAM/P6_ present features of progenitor cells with disturbed metabolism and could serve as appropriate model for in vitro investigation of age‐dependent osteoporosis.

## 1 INTRODUCTION

Osteoporosis represents the most common bone disease in elderly patients of both sexes and all races, occurring worldwide.^1^ Moreover, its incidents are more frequent in well‐developed and ageing societies.^2^ Notably, more than 200 million citizens worldwide currently suffer from osteoporosis, and ~8.9 million fractures are caused by osteoporosis‐related fractures.^3^ Furthermore, the specialists estimate that within 50 years, osteoporosis will reach the scale of global epidemy. The sudden termination of physical activity of osteoporotic patients, especially those suffering from bone fractures, becomes an even higher sociological cost that eliminates them from social life.^4^ Therefore, the investigation of novel concepts improving knowledge about the molecular mechanism of osteoporosis occurrence is critical for developing new therapeutic strategies and very much needed.

Osteoporosis is characterized by low bone mass as a result of impaired bone mineralisation, leading to reduced bone mechanical properties, increasing fracture risk.^5^ The pathophysiological mechanism of osteoporosis includes the deterioration of bone metabolisms. It is a consequence of several factors, including the advantage of bone resorption over bone formation process. The impairment of bone remodelling is caused by an imbalance between osteoclasts and osteoblasts, that is between bone‐degrading and bone‐producing cells.^6^ The recruitment of osteoclasts and osteoblast at the bone remodelling site requires activation of the plethora of molecular signals including hormones, cytokines, growth factors and non‐coding RNAs, including long non‐coding RNA (lncRNA) and microRNA (miRNA), which mediates the interaction between bone cells and progenitor cells.^7^ Moreover, the recruitment of bone marrow stromal cells (BMSCs), which are a source of progenitor cells at the bone remodelling site, guarantees a supportive role during new bone formation.

Bone marrow‐derived stromal cells (BMSCs) are multipotent stem cells with self‐renewal capacity.^8^ The population of showing features of BMSCs was described for the first time by Alexander Friedenstein and colleagues.^9^, ^10^ Since that time, the knowledge regards BMSCs biology and nature are still extensively investigated.^8^, ^11^ According to the current statement of *International Society for Cellular Therapy* (ISCT) BMSCs as a mesenchymal stromal stem cell are characterized by: (a) expression of CD73, CD90 and CD150 and lack of expression of CD11b, CD14, CD19, CD34, CD45 and HLA‐DR molecules, (b) adhesion to the plastic surface of culture dish under standard culture condition and (c) possesses the ability for differentiation into chondrocytes, osteoblasts and adipocytes in vitro.^12^ The self‐renewal potential, associated with increased proliferative capacity, shed a promising light for various clinical application of BMSCs transplants. Numerous studies identified the molecular mechanisms of BMSCs, emphasizing their beneficial effects in the course of fractures bone regeneration.^13^, ^14^, ^15^ The progenitor cells of bone marrow express critical markers for new bone formation, which included alkaline phosphatase (ALP), bone morphogenetic protein 2/4 (BMP‐2/4), osteoprotegerin (OPG), receptor activator of nuclear factor B (RANK), RANK ligand (RANKL), osteocalcin (OCL), osteopontin (OPN), wingless‐type MMTV integration site family (Wnt) proteins and signalling through parathyroid hormone receptors.^16^, ^17^

The pro‐regenerative potential of BMSCs is also mediated by their paracrine activity and shedding the extracellular microvesicles (ExMV’s), which are particularly rich in growth factors, miRNAs or lncRNAs.^18^, ^19^, ^20^ Moreover, various miRNA and lncRNA have been shown recently to be involved in the mediation of balance between cell populations of osteoblast‐like or osteoclast‐like nature.^7^, ^21^ Recent data suggest that the immunomodulatory activity of BMSCs makes them an even more promising therapeutic tool in terms of cell‐based therapies in osteoporosis treatment.^22^

However, the metabolic imbalance associated with osteoporosis affects the activity of BMSCs. The cells are losing their valuable biological properties, such as proliferative activity and multipotency. Moreover, BMSCs isolated from osteoporotic patients show apoptotic phenotype and accumulation of oxidative stress factors, which seriously reduce their viability. BMSCs isolated from osteoporotic rat show increased expression of several markers related to adipogenesis and simultaneously reduced expression of master regulators essential for bone formation.^23^, ^24^ Thus, BMSCs are currently extensively investigated since understanding their molecular nature under osteoporosis might bring us closer to understanding the molecular mechanism involved in osteoporosis development.

For that reason, in this study, for the first time, we have isolated BMSCs derived from osteoporotic mice strain SAM/P6 (BMSC_SAM/P6_) and described it as a genuine and relevant in vitro model, allowing determination of the molecular basis of osteoporosis development Current models rely on BMSCs isolated from ovariectomized rats^23^, ^24^ or patients with osteoporosis^17^, ^25^, ^26^ However, still the molecular aspects of BMSCs cytophysiology affected by osteoporosis has not been fully elucidated. Here, we have characterized BMSC_SAM/P6_ proliferative and metabolic activity and determined the expression of common phenotype markers, critical for stemness. Moreover, using cytometric‐based tests, we have confirmed lowered metabolism of the cells, associated with depolarization of the mitochondrial membrane, intracellular accumulation of reactive oxygen species (ROS), accompanied with down‐regulation of mitofusin 1 (MFN‐1) protein expression in osteoporotic BMSCs compared with BMSCs isolated from healthy tissue. Additionally, we have evaluated the multipotency of BMSC_SAM/P6_ and determined the expression profile of bone‐related markers (lncRNA‐miRNA‐mRNA axis). The molecular pattern of miRNAs expression, for example miR‐21‐5p or miR‐124‐3p in osteoporotic murine BMSCs has not been previously evaluated by other authors. Moreover, the analysed miRNAs were referred to the expression of lncRNA (*DANCR1*) and mRNAs, including *Runx‐2* (runt‐related transcription factor 2), *Trap* (tartrate‐resistant acid phosphatase) or *Opn* (osteopontin). Obtained results were compared with BMSCs isolated from healthy BALB/c mice (BMSC_BALB/c_). Here, we characterized novel bone marrow multipotent stem cells that could be used in future research regarding osteoporosis, especially attributed to ageing.

## 2 MATERIALS AND METHODS

### 2.1 Isolation procedure and propagation of bone marrow‐delivered progenitor stem cells

The bone marrow‐derived stromal cells were isolated from long bones of mice collected from lower limbs. After removal, bones were washed twice in Hank's Balanced Salt Solution (HBSS) with 1% addition of antibiotics (P/S—penicillin and streptomycin). The distal parts of every bone were cut out. Following that, bone marrow was isolated by its flushing from the medullary canal with an insulin syringe U‐40 (29G X 1/2′′ needle) filled with HBSS as described previously.^27^ The cells were collected and centrifuged two times (300 × g, 4 min). Subsequently, the isolated cells were counted by Muse^®^ Count & Viability Kit (Merck®; cat. no.: MCH100102, Poznan, Poland). The procedure was carried out following protocol provided by the manufacturer. Further, the cells were inoculated on the 24‐well dishes at density 800 000 cells/well and suspended in 500 µL of complete growth medium (CGM), consisted of Ham's F‐12 Nutrient Mixture (F‐12) supplemented with 15% of foetal bovine serum (FBS) and 1% of antibiotics (P/S). The cultures were propagated in sterile conditions using CO_2_ incubator with constant parameters: 5% CO_2_, 37°C and 95% humidity. After 24 hours of culture, the media were removed and replaced by the fresh media in order to eliminate hematopoietic cell lineage.^28^ All reagents used for cell cultures (media, HBSS, antibiotics, FBS) were derived from Sigma‐Aldrich (Poznan, Poland).

During propagation culture condition, growth pattern, as well as cells morphology were monitored using Axio Observer A1‐inverted microscope (Zeiss, Oberkochen, Germany) and documented with Canon PowerShot digital camera (Woodhatch, UK). The photographs were taken under 100× and 400× magnification.

### 2.2 Analysis of BMSCs metabolic activity

The analysis of BMSC_BALB/c_ and BMSC_SAM/P6_ metabolic activity was carried out by the use of well‐established Alamar Blue test. After five days of cultures propagation, the cells were washed once with HBSS and 350 µL of CGM with 10% addition of resazurin dye solution (Tox8‐1KT, Sigma‐Aldrich, Munich, Germany) was added. Subsequently, the cultures were incubated for 2 hours in 37°C (5% CO_2_ and 95% humidity). After incubation, the supernatant was removed and transported to the 96‐well dish in six repetitions. The absorbance was measured at the wavelengths of 600 and 690 nm. The metabolic activity of BMSCs was calculated using formula: ΔA = A600nm – A690nm.

### 2.3 Immunocytochemical detection of CD44, CD73, CD90 and CD45

In order to characterize the isolated cells, surface markers typical for BMSCs were stained. After reaching ~80% of confluency, the cells were fixed with 4% PFA (paraformaldehyde) for 30 minutes at room temperature. Further, cultures were washed three times with HBSS and permeabilised with 0.2% PBS‐Tween solution with 10% addition of goat serum for 1 hour. Subsequently, specimens were washed 3 times with HBSS and incubated overnight at 4°C with primary antibodies: anti‐CD44 (hpa005785, Sigma‐Aldrich, Munich, Germany) in the dilution of 1:1000; anti‐CD73 (ab54217, Abcam, Cambridge, UK) in the dilution of 0.1 µg/100 µL; anti‐CD90 (ab92574, Abcam, Cambridge, UK) in the dilution of 1:100; and anti‐CD45 (sc‐53047, Santa Cruz Biotechnology, Dallas, Texas, USA) in the dilution of 1:100. Anti‐CD44, anti‐CD90 antibodies were produced in rabbit and anti‐CD73, anti‐CD45 antibodies were produced in mouse. After the overnight incubation, the specimens were washed 3 times with HBSS. Following washing, specimens were incubated for 1 hour at room temperature with secondary antibodies: anti‐mouse IgG—Atto 594 antibody produced in goat (76085, Sigma‐Aldrich, Munich, Germany) and anti‐rabbit IgG—Atto 594 antibody produced in goat (77671, Sigma‐Aldrich, Munich, Germany). The concentration of secondary antibodies was 1:1000. Finally, specimens were washed (as above) and fixed on slides using the mounting medium with DAPI (4′,6‐diamino‐2‐phenolindole) as a nuclear counterstain (Fluoroshield^TM^ with DAPI, Sigma‐Aldrich, Munich, Germany). The specimens were analysed using a confocal microscope (Leica TCS SPE, Leica Microsystems, KAWA.SKA Sp z o.o., Zalesie Górne, Poland). The microscopic images were obtained by application of maximum intensity projection (Z‐Project). The photographs were captured under 630× magnification. Obtained signals after cell surface antigens staining were measured using Fiji is just ImageJ and Pixel Counter plugin (version 1.52n, Wayne Rasband, National Institutes of Health, USA). The differences between the amount of colour pixels in CD44, CD45, CD73 and CD90 staining were determined in three technical repetitions and using three different thresholds (29, 30 and 31) within ImageJ Software.

### 2.4 Chondrogenic, osteogenic and adipogenic differentiation of BMSCs

In order to prove the multipotent abilities of isolated BMSCs, chondrogenic, osteogenic and adipogenic differentiation of cultures were performed with differentiation media.

The chondrogenic medium was prepared using StemPro Osteocyte/Chondrocyte Differentiation Basal Medium (A10069‐01, Gibco, Life Technologies Corporation, USA) and StemPro Chondrogenesis Supplement (A10069‐01, Gibco, Life Technologies Corporation, USA) in the ratio of 10:1, respectively. The adipogenic medium was prepared using StemPro Adipogenesis Differentiation Basal Medium (A10410‐01, Gibco, Life Technologies Corporation, USA) and StemPro Adipogenesis Supplement (A10065‐01, Gibco, Life Technologies Corporation, USA) in the ratio of 10:1, respectively. The chondrogenic and adipogenic media were supplemented with 0.05% of gentamycin, according to the manufacturer's protocol. The fresh chondrogenic and adipogenic media were changed twice a week and maintained for 7 days.

The osteogenic medium was prepared using Minimum Essential Medium Eagle—Alpha Modification (MEM‐α), supplemented with osteogenic factors as was described previously^21^ : 50 µg/mL of ascorbic acid (Sigma‐Aldrich, Munich, German) and 10 nmol/L of β‐glycerol phosphate disodium salt hydrate (Sigma‐Aldrich, Munich, Germany). The fresh osteogenic medium was changed twice a week. The osteogenic conditions were maintained for 10 days. After the differentiation process, the cultures were collected for subsequent analyses.

### 2.5 Evaluation of BMSCs extracellular matrix composition and neutral lipids staining after differentiation conditions

The protocol of extracellular matrix staining was described previously.^21^, ^29^ Briefly, differentiated cultures of BMSCs were fixed with 4% paraformaldehyde (PFA) for 15 minutes at room temperature and stained with specific dyes. Safranin‐O was used for proteoglycans detection and Alizarin Red for calcium deposits detection. Obtained specimens were analysed using Axio Observer A1‐inverted microscope (Zeiss, Oberkochen, Germany) and documented with Canon PowerShot digital camera (Woodhatch, UK). The photographs of visualized proteoglycan and calcium deposits were taken under 100× magnification. In order to visualize the neutral lipid droplets, HCS LipidTOX Green Neutral Lipid Stain was used according to manufacturer's protocol (H34475, Sigma‐Aldrich) and observed under a confocal microscope (Leica TCS SPE, Leica Microsystems, KAWA.SKA Sp z o.o., Zalesie Górne, Poland). The photographs of visualized neutral lipid droplets were taken under 630× magnification. Obtained signals were measured using Fiji (ImageJ) and Pixel Counter plugin (version 1.52n, Wayne Rasband, National Institutes of Health, USA) as described previously.^30^ The differences between the amount of colour pixels were determined in three technical repetitions and using three different thresholds (osteogenesis/chondrogenesis—239, 240 and 241; adipogenesis—48, 50 and 51) within ImageJ Software.

### 2.6 Immunocytochemical detection of RUNX‐2, OPN and TRAP

The procedure of RUNX‐2, OPN and TRAP protein staining using confocal microscopy was mentioned in Section 2.3. and was described previously in detail.^21^, ^31^ The used primary antibody were anti‐RUNX‐2 antibody (F‐2) produced in mice (sc‐390351, Santa Cruz Biotechnology, Dallas, Texas, USA) diluted to concentration 1:50 in HBSS; anti‐OPN antibody produced in rabbit (ab8448, Abcam, Cambridge, UK) diluted to concentration 1:100 in HBSS; and anti‐TRAP antibody (D‐3) mouse monoclonal IgG1 (sc‐376875, Santa Cruz Biotechnology, Dallas, Texas, USA) diluted to concentration 1:50 in HBSS. Following incubation with primary antibody, samples were washed three times with HBSS and incubated with secondary antibody (anti‐mouse or anti‐rabbit IgG—Atto 594 antibody produced in goat, Sigma‐Aldrich, Munich, Germany) for 1 hour at room temperature. The concentration of secondary antibodies was 1:1000. The analysis of the samples was described in Section 2.3. The differences between the amount of colour pixels in RUNX‐2, OPN and TRAP staining were determined in three technical repetitions and using three different thresholds (29, 30 and 31) within ImageJ Software.

### 2.7 Analysis of BMSCs apoptosis profile and viability

Analysis of apoptosis profile and viability in BMSC cultures was carried out using the Muse™ Annexin V & Dead Cell Kit (Merck®; cat. no.: MCH100105, Poznań, Poland). The procedure was performed according to the producer's protocol after five days of culture propagation. Before the test cultures were trypsinised (StableCell Trypsin, Sigma‐Aldrich, Munich, Germany) and suspended in 100 µL of phosphate‐buffered saline (PBS) supplemented with 1% of FBS. Further, 100 µL of Muse™ Annexin V & Dead Cell Reagent was added to the cells and they were incubated at room temperature for 20 minutes. The reagent provided by the manufacturer consisted of two dyes: Annexin V and 7‐Aminoactinomycin D (7‐AAD). The apoptosis profile and percentage of viable cells were determined by the use of Muse^TM^ Cell Analyzer. Each analysis was performed in triplicate. The gating procedure of cells’ populations was based on the positive and negative controls.^32^, ^33^, ^34^

### 2.8 Analysis of BMSCs caspase activation profile

The activation of caspases was determined using the Muse™ MultiCaspase Kit (Merck®; cat. no.: MCH100109, Poznań, Poland). The whole procedure was performed accordingly to manufacturer's protocol and our previous experiment.^35^ Briefly, Caspase buffer was diluted 10× in DEPC‐treated water and the MultiCaspase Reagent Stock Solution was diluted in 50 µL of DMSO. Other reagents were prepared for the analysis as it was described elsewhere.^35^ According to manufacturer instructions, the analysis of caspases activation was based on membrane permeable VAD‐peptide that can detect multiple caspases for example caspase 1, −3, −4, −5, −6 −7, −8 and −9. Stained samples were incubated for 30 min (37°C, 95% humidity, 5% CO_2_) and 150 µL of Muse^TM^ 7‐AAD Working Solution was added in order to detect dead cells. The caspases activity profile was measured using Muse^TM^ Cell Analyzer. Each analysis was performed in triplicate. The gating procedure of cells’ populations was based on the positive and negative controls.^32^, ^33^, ^34^

### 2.9 Analysis of BMSCs reactive oxygen species activation

The analysis of reactive oxygen species activation (ROS) was measured using the Muse™ Oxidative Stress Kit (Merck^®^; cat. no.: MCH100111, Poznań, Poland). The staining procedure was performed accordingly to producer's protocol and described previously.^32^ Briefly, after trypsinisation 10 µL of cells were added to 190 µL of Muse Oxidative Stress Working Solution and incubated 30 minutes in 37°C. The staining reagent was provided by manufacturer and based on dihydroethidium (DHE), which is widely used for ROS detection in many cell cultures.^36^ Then, the oxidative stress was measured using Muse^TM^ Cell Analyzer. Each analysis was performed in triplicate. The gating procedure of cells’ populations was based on the positive and negative controls.^32^, ^33^, ^34^

### 2.10 Analysis of BMSCs mitochondrial membrane depolarisation status

The measurements of mitochondrial membrane depolarisation were determined using the Muse™ MitoPotential Kit (Merck^®^; cat. no.: MCH100110, Poznań, Poland). Firstly, Muse™ MitoPotential working solution was prepared by diluting MitoPotential Dye with 1X Assay Buffer in concentration of 1:1000. The MitoPotential Dye is a cationic, lipophilic solution that detects the changes in mitochondrial membrane potential and was provided by the manufacturer. Further, 95 µL of prepared dye was added to 100 µL of the cells and incubated 20 minutes in 37°C. After the incubation, 5 µL of Muse™ 7‐AAD was added to the samples in order to stain dead cells. After 5 minutes of incubation in room temperature, the depolarisation of cells’ mitochondrial membrane was measured using Muse^TM^ Cell Analyzer. Each analysis was performed in triplicate. The gating procedure of cells’ populations was based on the positive and negative controls.^32^, ^33^, ^34^

### 2.11 Analysis of MFN‐1 and PINK1 protein expression in BMSCs

In order to determine the extracellular level of accumulated proteins, the cell cultures were lysed by the use of ice‐cold RIPA buffer supplemented with 1% of protease and phosphatase inhibitor cocktail (Sigma‐Aldrich, Munich, Germany). The Bicinchoninic Acid Assay Kit was used to determine the amount of isolated protein (Sigma‐Aldrich, Munich, Germany). The samples containing 8 µg of protein were mixed with 4× Laemmli loading buffer and incubated at 95°C for 5 min in T100 Thermal Cycler (Bio‐Rad, Hercules, CA, USA). The electrophoresis reaction (SDS‐PAGE) was performed in 11% sodium dodecyl sulphate‐polyacrylamide gel for 90 minutes at 100V using Mini‐PROTEAN Tetra Vertical Electrophoresis Cell (Bio‐Rad, Hercules, CA, USA). Subsequently, the samples were transferred into polyvinylidene difluoride membrane (PVDF) using the Mini Trans‐Blot® system (Bio‐Rad, Hercules, CA, USA) for 1h at 100V. Then, membranes were blocked by the use of 5% skim milk powder in TBST buffer for 1h and then incubated overnight at 4°C with primary antibodies. The used primary antibodies were anti MFN‐1 antibody produced in rabbit (orb11040, Biorbyt) in dilution 1:500 and anti PINK1 antibody produced in rabbit (orb331223, Biorbyt) in dilution 1:250. The reference was anti β‐ACT antibody produced in rabbit (a5441, Sigma‐Aldrich) in dilution 1:2500. Membranes were washed 5 times for 5 min in TBST buffer. The incubation with secondary antibodies was performed for 1h at 4°C (Goat Anti‐Rabbit IgG Antibody in dilution 1:2500, ap156p, Sigma‐Aldrich). Subsequently, membranes were washed 5 times as described previously and analysed using Bio‐Rad ChemiDoc™ XRS system (Bio‐Rad, Hercules, CA, USA). The chemiluminescent signal was detected by the use of DuoLuX^®^ Chemiluminescent and Fluorescent Peroxidase (HRP) Substrate (Vector Laboratories). The signal intensity and molecular weight of detected proteins was analysed using Image Lab™ Software (Bio‐Rad, Hercules, CA, USA).

### 2.12 Analysis of mRNA, miRNA and lncRNA expression

The transcripts levels for selected mRNA, miRNA and lncRNA were evaluated using reverse transcription quantitative polymerase chain reaction (RT‐qPCR). After experiment, cultures were homogenized using 1 mL of Extrazol^®^ (Blirt DNA, Gdańsk, Poland). The isolation procedure of RNA was performed accordingly to manufacturer's protocol. After isolation, total RNA was diluted in molecular grade water (Sigma‐Aldrich, Poznan, Poland). The quantity and purity was evaluated spectrophotometrically at 260 and 280 nm wavelength (Epoch, BioTek, Bad Friedrichshall, Germany). The gDNA was digested by total RNA treatment with DNase I using PrecisionDNAse Kit (Primerdesign, BLIRT SA, Gdańsk, Poland). Synthesis of cDNA was performed from 190 ng of isolated RNA applying Tetro cDNA Synthesis Kit (Bioline Reagents Limited, London, UK). The procedure was carried out accordingly to manufacturers’ protocol in T100 Thermal Cycler (Bio‐Rad, Hercules, CA, USA). Moreover, Mir‐X^TM^ miRNA First‐Strand Synthesis Kit (Takara Clontech Laboratories, Biokom, Poznań, Poland) was used to evaluate non‐coding RNA levels. For this purpose, 150 ng of RNA was used and the procedure was carried out according to the manufacturer's protocol.

RT‐qPCR was performed with SensiFAST SYBR^®^&Fluorescein Kit (Bioline Reagents Ltd., London, UK) in CFX Connected Real‐Time PCR Detection System (Bio‐Rad, Hercules, CA, USA). The reaction for mRNA and lncRNA was carried out according to presented conditions: 95°C for 2 minutes (initial denaturation), then 45 cycles at 95°C for 5 s, annealing for 10 s and 72°C for 5 s (elongation). The melting curve using a gradient protocol (65°C–95°C with heating rate 0.2°C/s). For miRNA levels detection, all reaction conditions maintain the same; however, annealing temperature was always 58.8 °C. All reactions were performed in at least three repetitions. Relative values of transcripts were calculated using RQ_MAX_ algorithm and presented in the graphs after conversion into log2 scale. The transcripts levels for mRNA and lncRNA were normalized to the housekeeping gene *Gapdh* (glyceraldehyde 3‐phosphatehydrogenase) and *B2m* (beta‐2‐microglobulin). The transcript levels for miRNA were calculated in relation to a snU6 gene. The list of used primers are enclosed in Table 1.

**TABLE 1:** The list of primers used for RT‐qPCR

Gene	Primer Sequence 5′‐3′	Annealing [°C]	Amplicon length [bp]	Accesion no.
Igf‐1	F:AGAGCCTGCGCAATGGAATA	58,8	152	NM_010512.5
	R:TGCTGATTTTCCCCATCGCT			
Bcl‐2	F:ATCGCCCTGTGGATGACTGAG	58,8	129	NM_000633.2
	R:CAGCCAGGAGAAATCAAACAGAGG			
Bax	F:ACCAAGAAGCTGAGCGAGTGTC	58,8	414	NM_001291428.1
	R:ACAAAGATGGTCACGGTCTGCC			
Mmp‐9	F:GATGCCAACCTCCTCAACGA	60	211	NM_053056.2
	R:GGAAGCGGTCCAGGTAGTTC			
Runx‐2	F:TCCGAAATGCCTCTGCTGTT	58,8	130	NM_001271630.1
	R:GCCACTTGGGGAGGATTTGT			
Coll‐1	F:CAGGGTATTGCTGGACAACGTG	61,4	107	NM_007742.4
	R:GGACCTTGTTTGCCAGGTTCA			
Opn	F:AGACCATGCAGAGAGCGAG	57,3	340	NM_001204203.1
	R:GCCCTTTCCGTTGTTGTCCT			
Ocl	F:GGTGCAGACCTAGCAGACACCA	57	100	NM_001032298.3
	R:CGCTGGGCTTGGCATCTGTAA			
Opg	F:AGCCACGCAAAAGTGTGGAA	58,8	149	NM_008764.3
	R:TCCTCTCTACACTCTCGGCA			
Trap	F:GTCTCTGGGGGACAATTTCTACT	60	241	XM_006509945.3
	R:GTTTGTACGTGGAATTTTGAAGC			
Rankl	F:ACGCAGATTTGCAGGACTCGAC	58,8	493	NM_011613.3
	R:TTCGTGCTCCCTCCTTTCATC			
Gapdh	F:GTCAGTGGTGGACCTGACCT	58,8	256	NM_001289746.1
	R:CACCACCCTGTTGCTGTAGC			
DANCR1	F:GCCACTATGTAGCGGGTTTC	58,8	129	NR_024031.2
	R:ACCTGCGCTAAGAACTGAGG			
miR‐7a‐5p	TGGAAGACTAGTGATTTTGTTGT	58,8	‐	MIMAT0000677
miR‐17‐5p	CAAAGTGCTTACAGTGCAGGTAG	58,8	‐	MIMAT0000649
miR‐21a‐5p	TAGCTTATCAGACTGATGTTGA	58,8	‐	MIMAT0000530
miR‐124‐3p	TAAGGCACGCGGTGAATGCC	58,8	‐	MIMAT0000134
miR‐145‐5p	GTCCAGTTTTCCCAGGAATCCCT	58,8	‐	MIMAT0000437
miR‐203a‐3p	GTGAAATGTTTAGGACCACTAG	58,8	‐	MI0000283
miR‐223‐3p	TGTCAGTTTGTCAAATACCCCA	58,8	‐	MIMAT0000280

### 2.13 Statistical analyses

Experimental values are presented as means of obtained from at least three technical repetitions and they supplemented with standard deviation (±SD). The statistical calculations and data presentation was done with GraphPad Prism 5 (GraphPad Software, San Diego, CA, USA). The data were analysed using Student's *t* test. Differences were considered as statistically significant at *P* < .05. Significant differences between groups were indicated with asterisks: * *P* < .05, ** *P* < .01, *** *P* < .001. Non‐significant differences were marked as *ns*.

## 3 RESULTS

### 3.1 Characterization of isolated BMSCs—growth pattern, metabolic activity and multipotency

**FIGURE 1:**
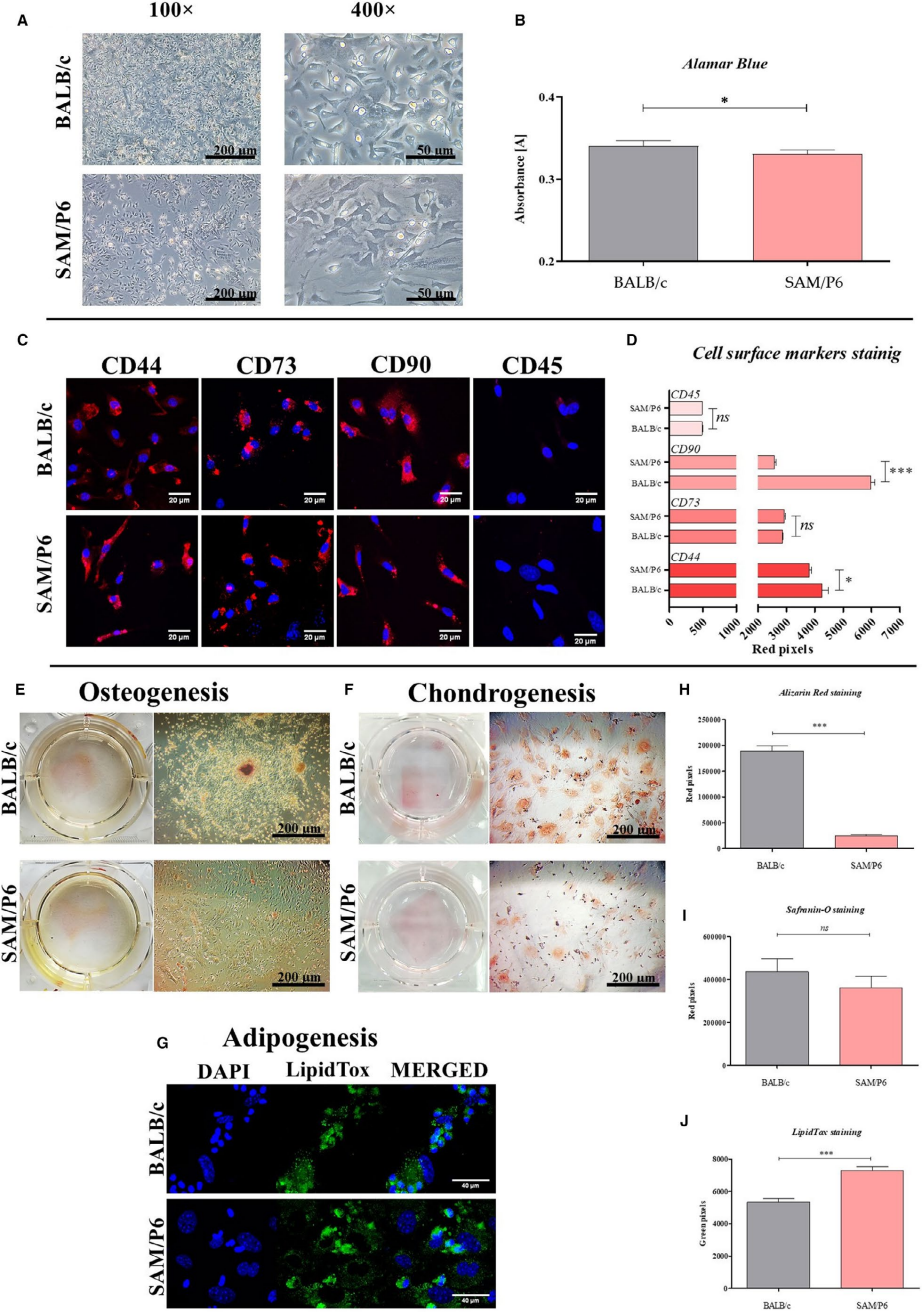
Characterization of BMSCs: The inverted light microscope images of BMSC_BALB/c_ and BMSC_SAM/P6_. The photographs were captured under 100‐ and 400‐fold magnification. The scale bars are equal 200 µm and 50 µm, respectively (A). The results of metabolic assay (Alamar Blue) determined for both tested population of BMSCs (B). The visualization of cells’ surface markers: CD44, CD73, CD90 and CD45. The microphotographs were taken using confocal microscope under 630× magnification. The scale bar is equal 20 µm (C). The comparative analysis based on staining intensity of surface markers presented as grouped bar graph (D). The images of BMSCs differentiated under osteogenic (E), chondrogenic (F) and adipogenic conditions (G). The photographs of cultures that undergo chondro‐ and osteogenesis were taken by the use of inverted light microscope under 100‐fold magnification, and the scale bar is equal 200 µm. The photographs of cultures that undergo adipogenesis were taken using confocal microscopy in order to visualize cells’ nuclei (DAPI) and lipid droplets (LipidTox). The confocal images were captured under 630‐fold magnification, and the scale bar is equal 40 µm. The stainings intensity measured in differentiated cultures was presented as bar graphs (H, I and J). Significant differences between groups are indicated with asterisks: * *P* < .05, ** *P* < .01, *** *P* < .001. Non‐significant differences are marked as *ns*

Isolated BMSCs, derived both from SAM/P6 and BALB/c mice, were successfully cultured in the monolayer system in sterile plastic dishes and maintained in the CO_2_ incubator with constant conditions: 37°C, 5% CO_2_ and 95% of humidity (Figure 1A). The morphology and growth pattern of BMSC cultures were characteristic for heterogenic population of multipotent stromal cells, with the predominant presence of fibroblast‐like, spindle‐shaped cells. The primary cultures of BMSC_SAM/P6_ had lowered confluency when compared to BMSC_BALB/c_, what was also reflected by their decreased proliferative activity. The metabolic activity of isolated BMSCs, measured by the use of Alamar Blue assay, showed that BMSC_SAM/P6_ metabolic rate was reduced (*P* <.05), and culture growth was impeded (Figure 1A and B).

Immunocytochemical staining showed that BMSC_SAM/P6_ and BMSC_BALB/c_ expressed typical cell surface confirming their mesenchymal origin (CD44, CD73 and CD90) and did not express CD45 characteristic for haematopoietic cells (Figure 1C). Importantly, the expression of CD44 and CD90 in BMSC_SAM/P6_ decreased (*P* < .05 and *P* < .001, respectively), compared with BMSC_BALB/c_ (Figure 1D).

After reaching around 80% of confluency, the cultures were differentiated under chondrogenic, osteogenic and adipogenic conditions (Figure 1E, F and G). When the differentiation process was finished, the extracellular matrix (ECM) formed in cultures was stained by specific dyes in order to analyse the amount of proteoglycans deposits (Safranin‐O staining), calcium deposits (Alizarin Red staining) and lipid droplets (LipidTox staining). No differences were noticed in the potential of both, BMSC_SAM/P6_ and BMSC_BALB/c,_ to differentiate into cartilage tissue (Figure 1F and I). However, the analyses of ECM composition indicated on lower potential (*P* < .001) of BMSC_SAM/P6_ to differentiate into bone tissue, compared with BMSC_BALB/c_ (Figure 1E and H). Simultaneously, BMSC_SAM/P6_ presented increased potential (*P* < .001) for lipid droplets formation and accumulation, when compared to BMSC_BALB/c_ (Figure 1G and J).

### 3.2 The BMSCs ultrastructure and expression of osteogenic markers

**FIGURE 2:**
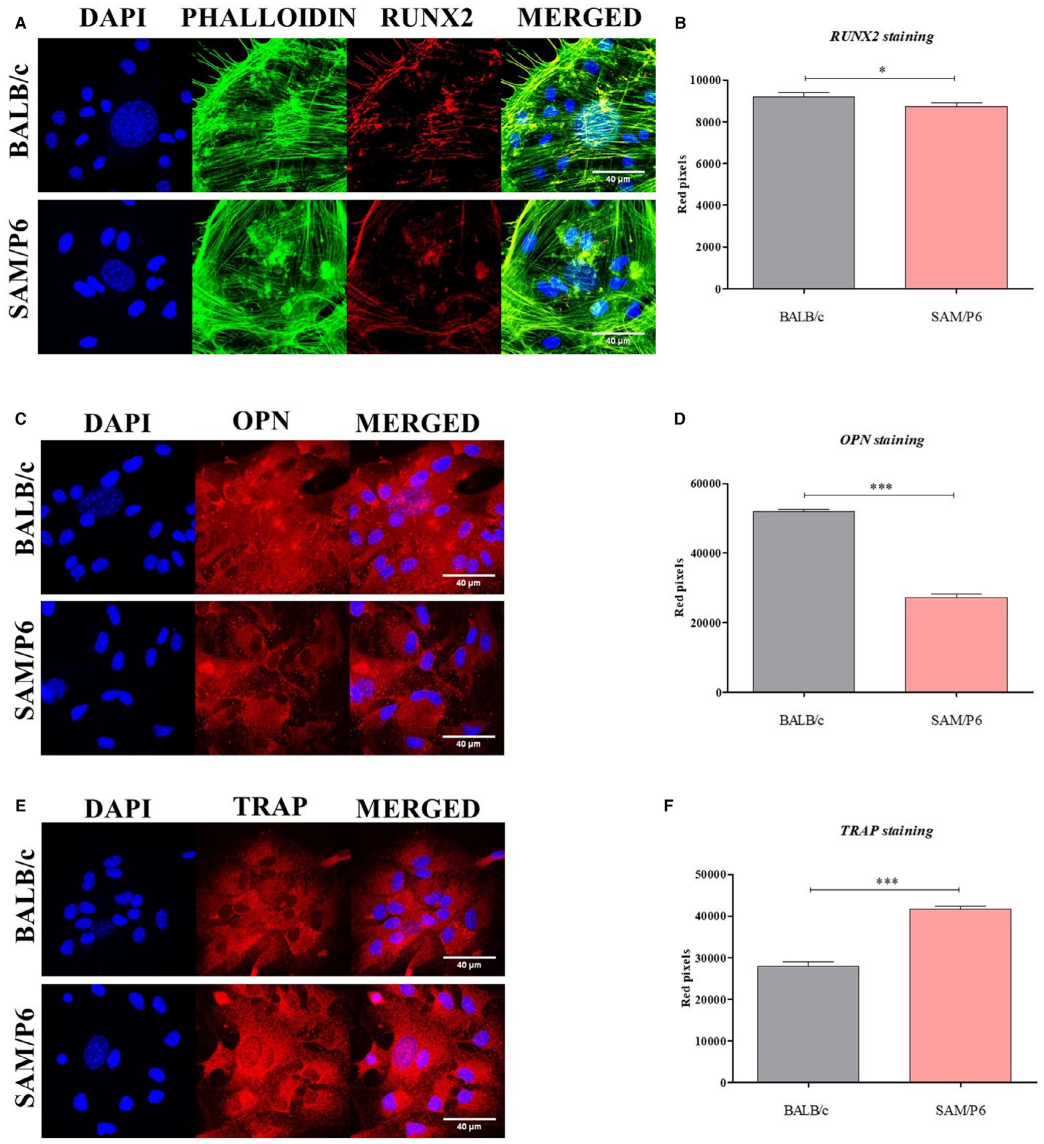
The morphology of BMSCs and expression of important osteogenic markers. The images show representative photographs (Z‐stacks) of: cells’ nuclei (DAPI) with actin cytoskeleton (Phalloidin) and co‐localisation of RUNX‐2 protein (A); cell's nuclei with co‐localisation of OPN protein (C); cells’ nuclei with co‐localisation of TRAP protein (E). The photographs were taken under 630‐fold magnification. The scale bar is equal to 40 µm. Moreover, the staining intensity (amount of red pixels) of visualized RUNX‐2, OPN and TRAP proteins were analysed and presented as bar graphs (B, D and F). Significant differences between groups are indicated with asterisk: *P < .05, **P < .01, ***P < .001. Non‐significant differences are marked as *ns*

Confocal imaging of cultures showed that the cytoskeleton network in BMSC_SAM/P6_ is less developed than in BMSC_BALB/c_. Additionally, the formation of cytoplasmic projections was less visible in BMSC_SAM/P6_ than in BMSC_BALB/c_. Poorly established actin cytoskeleton and intracellular connections influenced decreased confluency (cell to cell contact) in BMSC _SAM/P6_ cultures (Figures 1A and 2A). However, significant differences were noticed in the expression of osteogenesis‐dependent proteins: RUNX‐2 (runt‐related transcription factor 2), OPN (osteopontin) and TRAP (tartrate‐resistant acid phosphatase). The BMSCs isolated from SAM/P6 mice were characterized by lowered expression of RUNX‐2 (*P* < .05) and OPN (*P* < .001) proteins, key factors regulating osteogenic potential of progenitor cells (Figure 2B and D). Simultaneously, the expression of an osteoclastic marker, that is TRAP was increased (*P* < .001) in BMSC_SAM/P6,_ while BMSC_BALB/c_ cultures showed reversed phenotype. (Figure 2F). Obtained results are consistent with decreased deposition of calcium in ECM formed by BMSC_SAM/P6_ (Figure 1E and H).

### 3.3 Decreased expression of osteogenic markers is characteristic for BMSC derived from mice with osteoporotic phenotype

**FIGURE 3:**
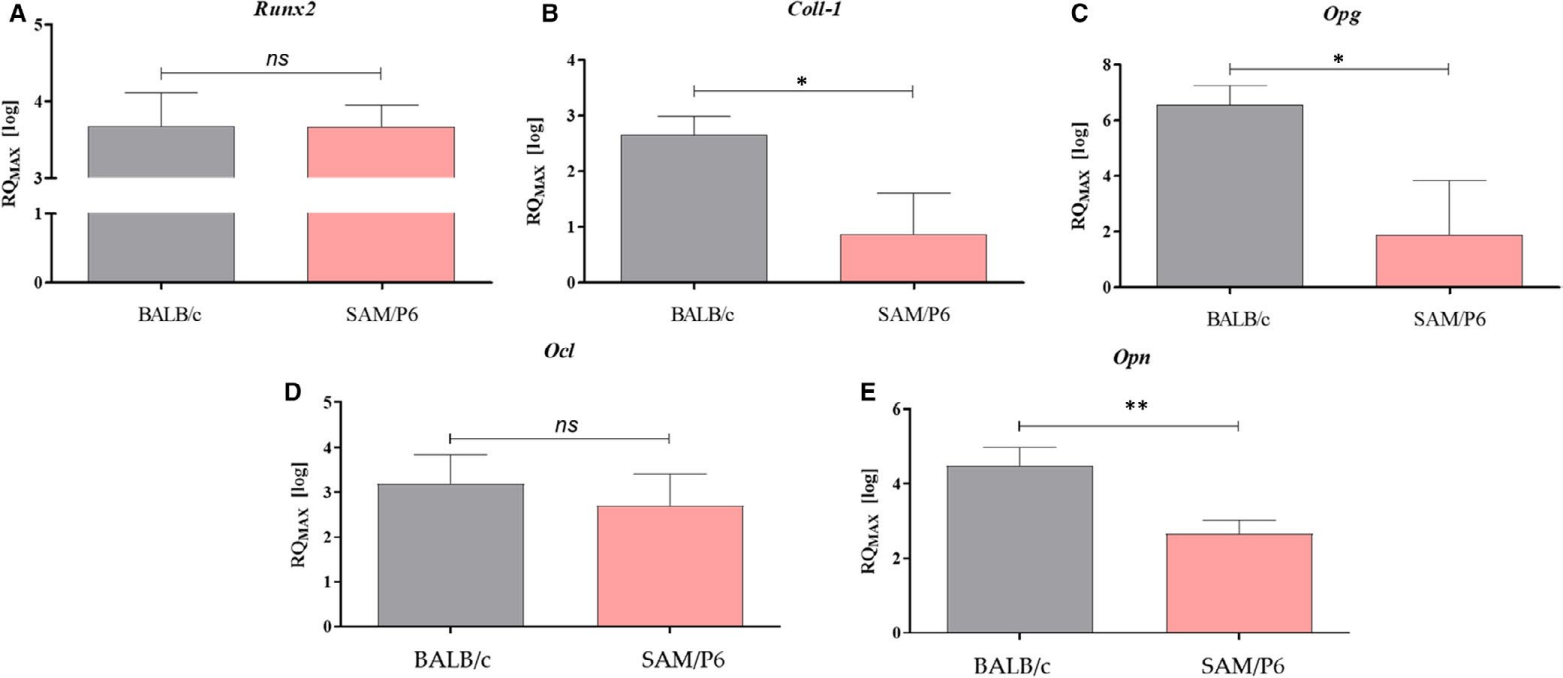
The analysis of genes’ transcriptomes (mRNA) associated with osteogenesis and bone homeostasis. The measurements were performed with RT‐qPCR technique, calculated using RQMAX method and presented in a log scale. The analysed targets were Runx‐2 (A); Coll‐1 (B); Opg (C); Ocl (D) and Opn (E). Significant differences between groups are indicated with asterisk: *P < .05, **P < .01, ***P < .001. Non‐significant differences are marked as *ns*

The analysis performed by the use of RT‐qPCR technique indicated that BMSC_SAM/P6_ are characterized by the reduced level of transcripts associated with proper osteogenesis and bone homeostasis. It has been shown that BMSC_SAM/P6_ had decreased mRNA level of *Coll‐1* (*P* < .05; collagen type 1), *Opg* (*P* < .05; osteoprotegrin) and *Opn* (*P* < .01; osteopontin) (Figure 3B, C and E). Moreover, the tendency of the expression of *Runx‐2* (runt‐related transcription factor 2) and *Ocl* (osteocalcin) in BMSCs indicated on the osteoporotic phenotype of BMSCs derived from SAM/P6 mice strain (Figure 3A and D).

### 3.4 Increased levels of Trap and osteogenesis‐dependent non‐coding RNAs distinguish BMSC derived from osteoporotic mice

**FIGURE 4:**
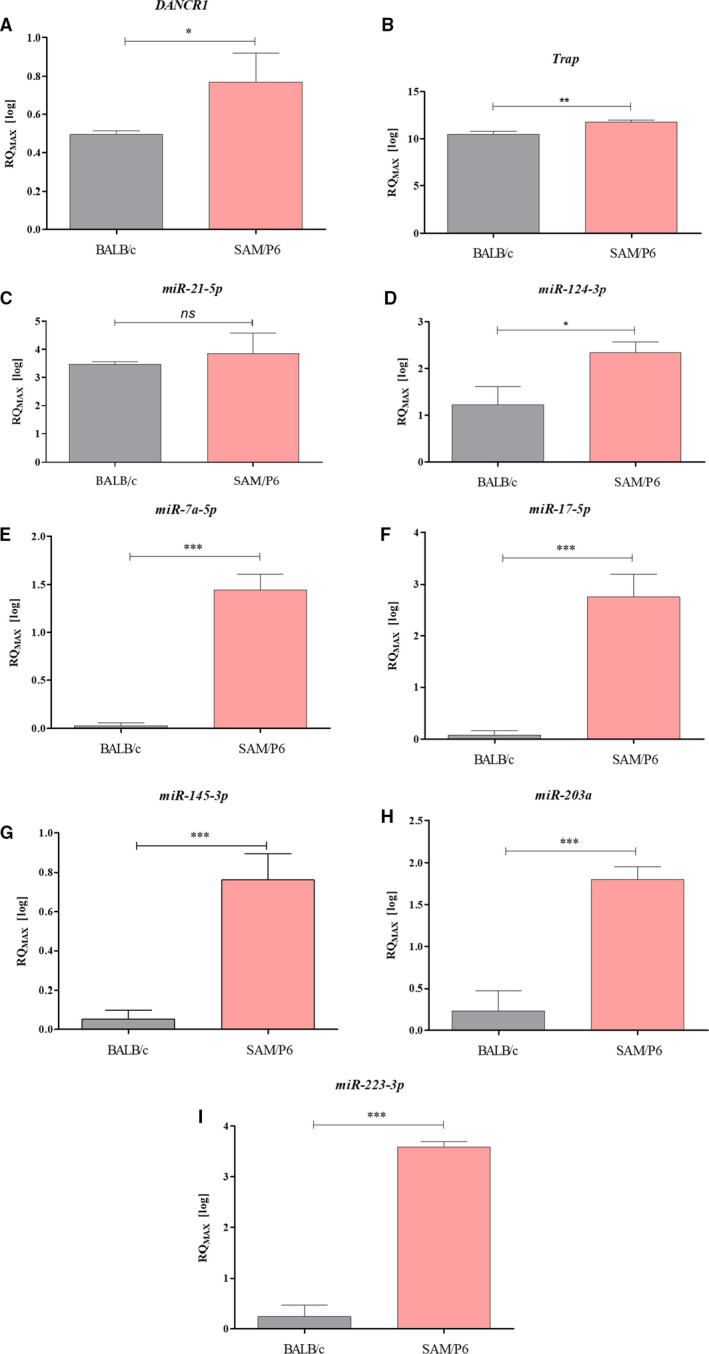
The analysis of genes’ transcriptomes (mRNA and non‐coding RNA) associated with osteoporosis and bone loss. The measurements were performed with RT‐qPCR technique, calculated using RQMAX method and presented in a log scale. The analysed targets were DANCR1 (A); Trap (B); miR‐21‐5p (C); miR‐124‐3p (D); miR‐7a‐5p (E); miR‐17‐5p (F); miR‐145‐3p (G); miR‐203a (H) and miR‐223‐3p (I). Significant differences between groups are indicated with asterisk: *P < .05, **P < .01, ***P < .001. Non‐significant differences are marked as *ns*

The RT‐qPCR analysis of mRNAs and non‐coding RNAs associated with osteoclastogenesis and bone loss confirmed the osteoporotic phenotype of BMSC_SAM/P6_ cultures. The expression level of non‐coding *DANCR1* (differentiation antagonizing non‐protein coding RNA 1) was significantly up‐regulated (*P* < .05) in BMSC_SAM/P6_ (Figure 4A). Moreover, it has been shown that the expression of *Trap* (tartrate‐resistant acid phosphatase) in BMSC_SAM/P6_ was significantly elevated (*P* < .01), compared with BMSC_BALB/c_ (Figure 4B). Furthermore, the analyses showed that the levels of typical miRNAs, characteristic for osteoporotic bone, were highly expressed in BMSC_SAM/P6_. We noted elevated levels of miR‐124‐3p (*P* < .05; Figure 4D), miR‐7a‐5p (*P* < .001; Figure 4E), miR‐17‐5p (*P* < .001; Figure 4F), miR‐145‐3p (*P* < .001; Figure 4G), miR‐203a (*P* < .001; Figure 4H) and miR‐223‐3p (*P* < .001; Figure 4I). The difference in the level of miR‐21‐5p, known from its dual activity towards bone cells, was insignificant (Figure 4C).

The expression profile of transcripts determined in BMSC_BALB/c_ and BMSC_SAM/P6_ was also presented as a heatmap in Supporting Information (Figure S1).

### 3.5 BMSC from osteoporotic mice are characterized by apoptotic phenotype and increased oxidative stress

**FIGURE 5:**
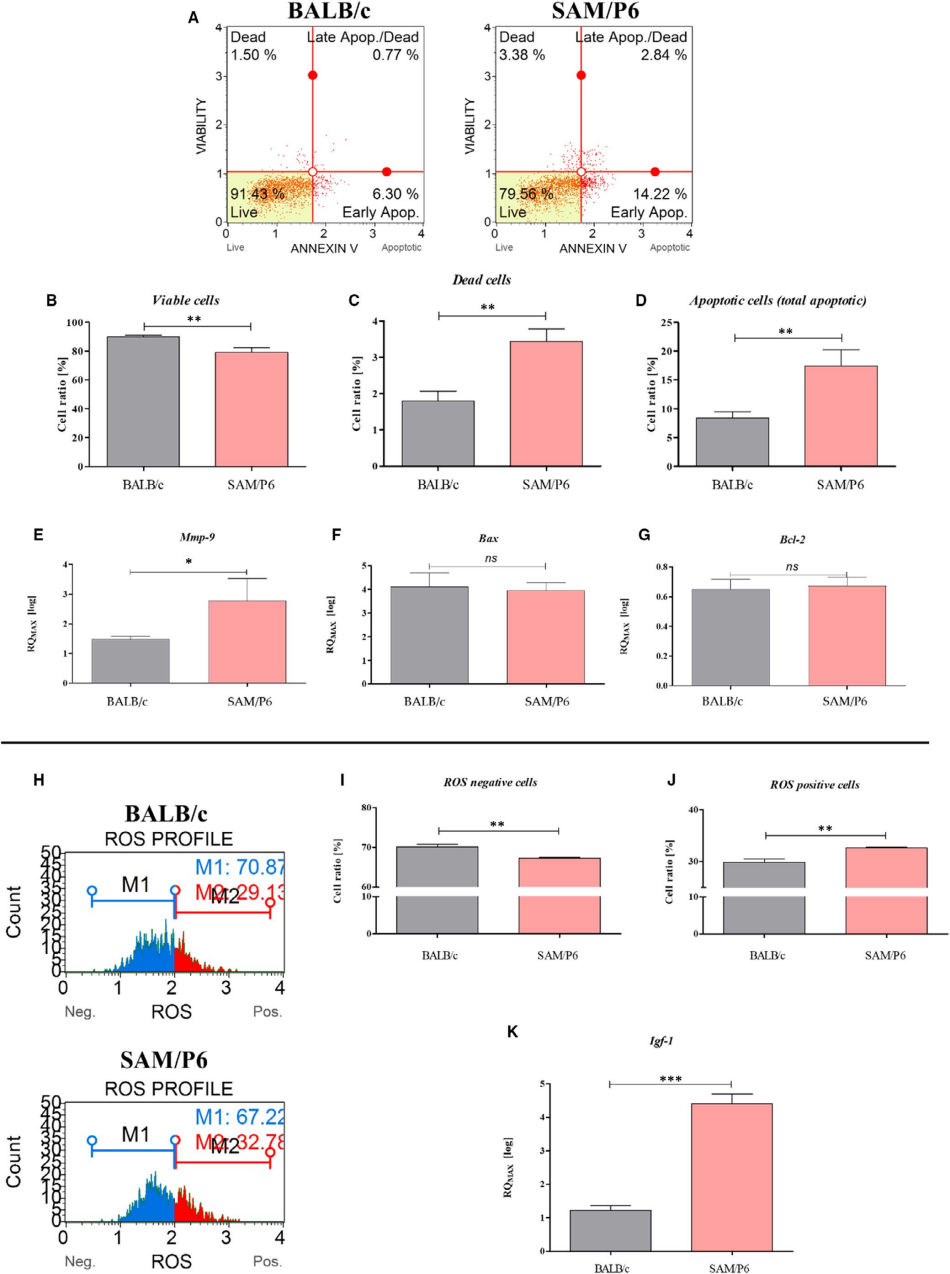
The BMSCs’ apoptosis profile and oxidative stress. The representative graphs of cells’ populations divided into four groups related to apoptosis profile (A): *live* (left bottom corner), *dead* (left upper corner), *early apoptosis* (right bottom corner) and *late apoptosis* (right upper corner). The comparison analysis of viable cells (B), dead cells (C) and apoptotic cells (D). The representative graphs of cells’ populations divided into two groups related to reactive oxygen species activation (H): ROS‐negative cells (blue colour) and ROS‐positive cells (red colour). The comparison analysis of ROS‐negative cells (I) and ROS‐positive cells (J). The mRNA expression of genes associated with cells’ viability and inflammation: *Mmp‐9* (E), *Bax* (F), *Bcl‐2* (G) and *Igf‐1* (K). The genes’ transcript levels were measured using RT‐qPCR technique, calculated with RQMAX method and presented in log scale. Significant differences between groups are indicated with asterisk: * *P* <.05, ** *P* <.01, *** *P* <.001. Non‐significant differences are marked as *ns*

It has been shown that BMSCs delivered from osteoporotic SAM/P6 mice were characterized by a lower ratio of viable cells (*P* < .01; Figure 5A and B) and a greater ratio of dead cells (*P* < .01; Figure 5A and C), compared with BMSCBALB/c. Moreover, BMSCSAM/P6 had a significantly greater ratio of cells that undergo apoptosis (*P* < .01; Figure 5A and D). The analysis performed by the use of RT‐qPCR technique showed no differences in the expression of important markers associated with programmed cell death, that is pro‐apoptotic *Bax* (Bcl‐2‐associated X protein) and anti‐apoptotic *Bcl‐2* (B‐cell lymphoma 2) (Figure 5F and G). However, BMSC_SAM/P6_ expressed more transcript for *Mmp‐9* (*P* < .05; metalloproteinase 9), an additional marker of apoptotic cells.^37^

The examination of oxidative stress in the isolated BMSCs indicated an increased accumulation of reactive oxygen species (ROS) in BMSC_SAM/P6_ (*P* < .01; Figure 5H and J). In turn, the reactivity of ROS in BMSC_BALB/c_ was lesser (*P* <.01; Figure 5H and I). Furthermore, it has been shown that BMSC_SAM/P6_ accumulates more transcripts for *Igf‐1* (*P* < .001; insulin‐like growth factor 1). In correspondence with increased ROS, the up‐regulated *Igf‐1* may be associated with the pro‐inflammatory activity of progenitor cells derived from bone marrow of SAM/P6 mice (Figure 5K).

### 3.6 Decreased viability of BMSC from osteoporotic mice can be a caspase‐independent process

**FIGURE 6:**
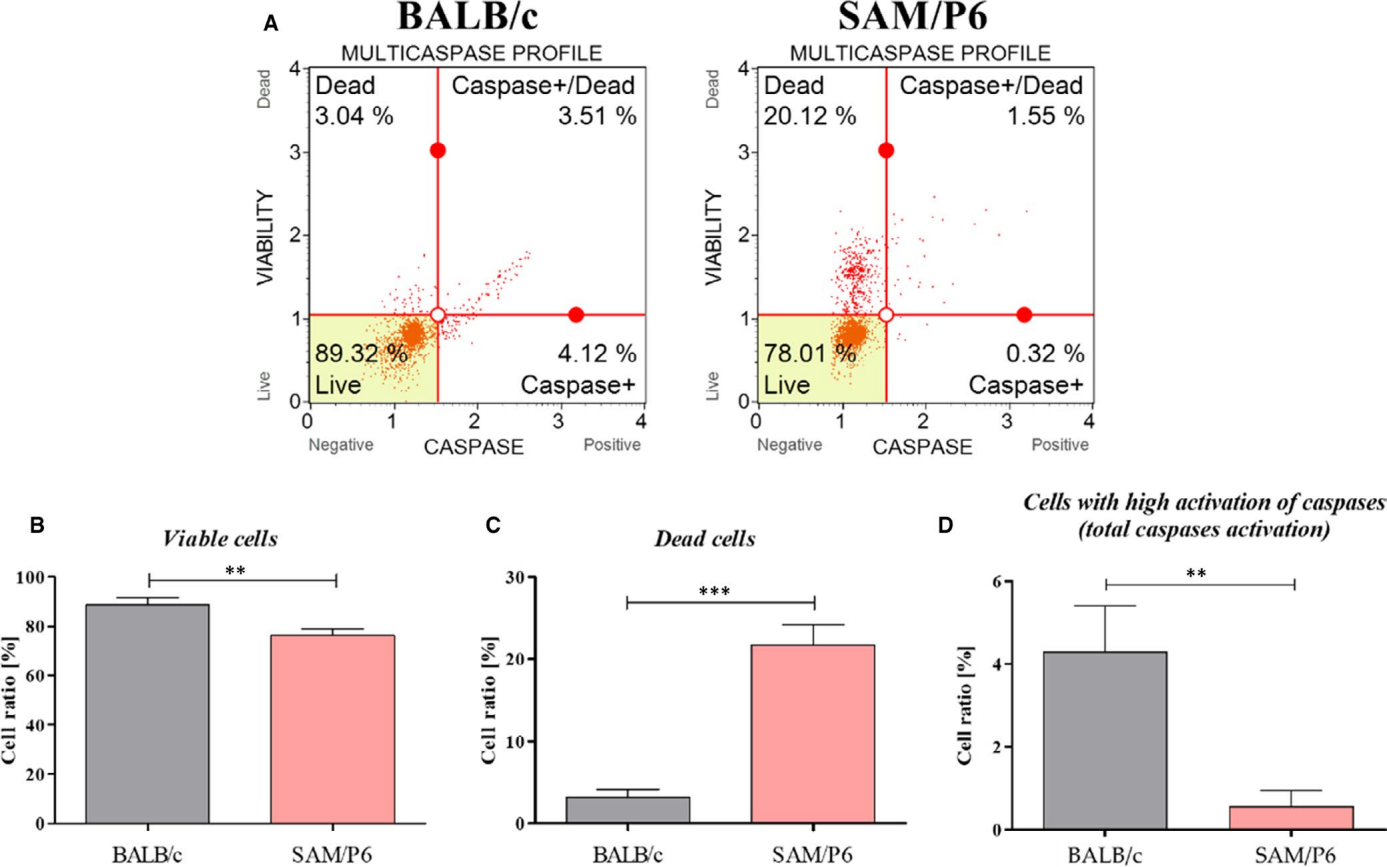
The activation of caspases in BMSCs. The representative graphs of cells’ populations divided into four groups (A): *live* (left bottom corner), *dead* (left upper corner), *viable cells with activated caspases* (right bottom corner) and *dead cells with activated caspases* (right upper corner). The comparison analysis of viable cells (B), dead cells (C) and cells with activated caspases (D). Significant differences between groups are indicated with asterisk: *P < .05, **P < .01, ***P < .001. Non‐significant differences are marked as *ns*

The flow cytometry‐based measurements showed that BMSC_SAM/P6_ were characterized by a lower cell ratio with activated caspases (*P* < .01; Figure 6A and D). However, the ratio of viable cells was decreased (*P* < .01) in BMSC_SAM/P6_ (Figure 6A and B) and ratio of dead cells was increased in BMSC_SAM/P6_ (*P* < .001; Figure 6A and C), compared with BMSC_BALB/c_. Obtained data suggested that the deteriorated viability of BMSC_SAM/P6_ is not a result of caspase‐dependent processes.

### 3.7 BMSCs with osteoporotic phenotype are characterized by mitochondrial membrane depolarisation and impaired dynamics of mitochondrial network

**FIGURE 7:**
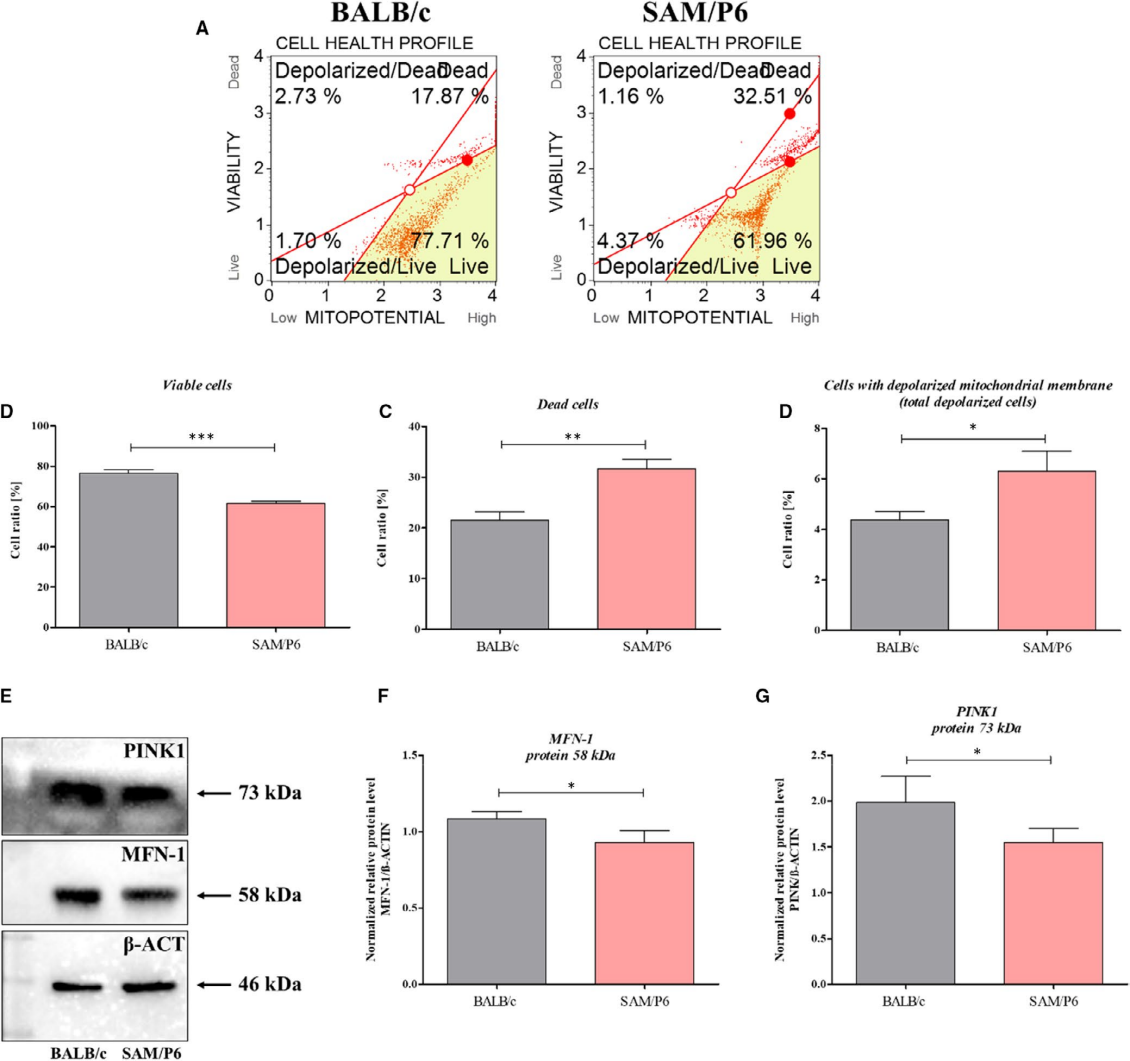
The electrostatic potential of mitochondrial membrane and mitochondrial dynamics in BMSCs. The representative graphs of cells’ populations divided into four groups (A): *live* (right bottom corner), *dead* (right upper corner), *depolarised/live* (left bottom corner) and *depolarised/dead* (left upper corner). The comparison analysis of viable cells (B), dead cells (C) and cells with depolarised mitochondrial membrane (D). The representative graph of Western blot (E). The mitochondrial dynamics was evaluated by protein level of MFN‐1 (F) and PINK1 (G). Significant differences between groups are indicated with asterisk: *P < .05, **P < .01, ***P < .001. Non‐significant differences are marked as *ns*

The mitochondrial membrane depolarisation status indicated on elevated cell ratio with depolarised mitochondrial membrane (*P* < .05) in BMSC isolated from SAM/P6 mice (Figure 7A and D). Thus, lowered activity of caspases and decreased viability of BMSC_SAM/P6_ may be associated with mitochondrial‐dependent pathway. The analysis of BMSC_SAM/P6_ viability, based on mitochondrial membrane potential, confirmed the increased death occurrence (*P* < .01) in those cultures (Figure 7A and C). Moreover, it has been shown that osteoporotic BMSC_SAM/P6_ were characterized by down‐regulation of protein levels for MFN‐1 (mitofusin 1; Figure 7E and F; *P* < .05) and PINK1 (PTEN‐induced kinase 1; Figure 7E and G; *P* < .05) compared with healthy BMSCBALB/c.

## 4 DISCUSSION

A new bone formation requires constant replenishment of the osteoblast from progenitor/stem cells mobilized from bone marrow. That cell lineage population needs to proliferate, differentiate and finally deposit a tissue‐specific extracellular matrix to create well‐developed and functional tissue. Numerous research groups worldwide study the biology of bone marrow‐derived stromal cells (BMSCs) in terms of osteoporosis development. Such studies are aimed to explore the cellular and molecular mechanisms involved in the progress of osteoporosis. So far, the ovariectomised rat derived BMSCs model has been extensively used for investigation of postmenopausal osteoporosis.^38^, ^39^ However, there are limited data regarding the biology of BMSCs characterized by senescence phenotype. Complete characterization of such population is needed to describe an appropriate model for investigating molecular and therapeutic targets of age‐related osteoporosis development.

Thus, for the first time in this study, we demonstrated and characterized a novel bone marrow‐derived stromal cells population isolated from senescence‐accelerated mouse stain prone 6 (BMSC_SAM/P6_) that resemble age‐dependent osteoporosis. Isolated cells exhibited senescence‐like phenotype, reduced proliferative and metabolic activity and seriously impaired multilineage differentiation potential comparing to the wild‐type BMSCs delivered from healthy BALB/c mice (BMSC_BALB/c_). The isolated BMSCs were characterized by typical markers such as CD44, CD73 and CD90, confirming their mesenchymal origin and indicating stemness. Moreover, obtained BMSCs showed lack of CD45 expression, which excludes their hematopoietic origin. Notably, BMSC_SAM/P6_ were characterized by reduced expression of CD44 and CD90, which are markers critical for multipotency of stromal cells and are related to progenitor cells’ proliferative activity.^40^ Obtained data are in line with previous research performed on BMSCs derived from patients with senile osteoporosis. The study showed that bone‐marrow progenitor cells of patients with age‐related osteoporosis are characterized by reduced proliferative activity, impaired phenotype, what affected disturbed recruitment and reduced regenerative potential.^26^ Numerous studies confirmed that increased patient age correlates with decreased beneficial properties of progenitor cells, namely “stemness” depending on lowered self‐renewal potential and causing defective extracellular matrix formation.^41^, ^42^

Moreover, progenitor cells with senescence phenotype exhibit seriously deteriorated multilineage differentiation potential, limiting their clinical application.^25^, ^43^ We have found that BMSC_SAM/P6_ were prone to accumulate lipid droplets and showed enhanced adipogenic differentiation. That indicates an advantage of adipogenic over osteogenic and/or chondrogenic‐like phenotype of BMSCs derived from patients suffering from senile osteoporosis. The loss of osteogenic potential of BMSC_SAM/P6_ was also related to decreased expression of RUNX‐2 and OPN expression and increased TRAP levels, characteristic for osteolytic cells.

Furthermore, cytometric‐based analyses of BMSCs’ viability indicated on the apoptotic profile of BMSC_SAM/P6_. Cells isolated from osteoporotic SAM/P6 mice were characterized by a lowered ratio of viable cells, simultaneously with a greater ratio of dead and apoptotic cells. Moreover, BMSC_SAM/P6_ expressed increased mRNA levels for *Mmp‐9* (gelatinase B), which can serve as an additional marker of cells that undergo apoptosis.^37^, ^44^ It has been shown that MMP‐9 may modulate the viability of cells, affecting pro‐apoptotic and anti‐apoptotic signals and proteins, such as BAX, BCL‐2, PARP or CASP‐3. However, MMP‐9 influence on the viability of BMSC has not been yet elucidated.

The decreased viability of BMSC_SAM/P6_ was also associated with the accumulation of reactive oxygen species (ROS) and corresponded to up‐regulated expression of *Igf‐1*. It has been proven that oxidative stress influences inflammatory cytokines like TNF‐α or interleukins, which orchestrated synergy plays a key role during intercellular redox state, leading to important alterations of the differentiation process and osteoporosis development.^45^, ^46^ Notably, BMSC_SAM/P6_ were characterized by significant depolarisation of the mitochondrial membrane, which also correlates with an apoptotic phenotype of BMSC_SAM/P6_. However, the analyses of caspases activation suggested that the apoptosis of BMSC_SAM/P6_ is not a caspase‐dependent process. It has been shown that caspase‐independent cell death (CICD) is an alternative pathway of programmed cell death. Although CICD proceeds in slower kinetics than classic apoptosis, it is related to large‐scale cytoplasmic vacuolisation, peripheral nuclear condensation and autophagosome accumulation.^47^, ^48^ Importantly, this mechanism of apoptosis is also reflected by depolarisation of the mitochondrial membrane. Nevertheless, more insightful examination needs to be performed to specify the apoptosis phenotype of BMSC_SAM/P6_ in detail.

The mitochondrial depolarization accompanied with accumulation of ROS, characteristic for BMSC_SAM/P6_ stands in line with lowered expression of mitofusin 1 (MFN‐1) and PTEN‐induced kinase 1 (PINK1) protein. It has been previously shown that MFN‐1 plays a vital role in the process of mitochondria fusion, thus maintaining proper mitochondrial dynamics.^49^, ^50^ Importantly, the impaired mitochondrial functionality caused by knockout of MFN‐1 in BMSCs has been previously associated with enhanced apoptosis and suppression of osteogenesis.^51^ Morover, previous studies have distinguished PINK1 as an essential regulator of mitochondria quality control, protecting against oxidative stress and disposal of damaged mitochondria.^52^, ^53^ Moreover, Feng et al (2021) have noted that PINK1 was highly engaged in the process of mitophagy in the rat BMSCs and proven its importance in maintaining the BMSCs’ stemness.^54^

Our previous study has shown that increased patient age corresponds with adipogenic and osteoclasts like phenotype of multipotent stromal cells, associated with high expression of p53.^43^ However, the mechanism of that phenomenon is still poorly investigated. For that reason, in this study, we investigated the expression of critical transcripts involved in regulating osteogenesis on mRNA, miRNA, and lncRNA. We have found, that BMSC_SAM/P6_ expressed higher osteoclasts related transcripts including *Trap* and long non‐coding RNA *DANCR1* together with elevated expression of miR‐7a‐5p, miR‐17‐5p, miR‐124‐3p, miR145‐3p, miR‐203a and miR‐223‐3p, which are critical for modulation of osteogenesis. *Trap* belongs to the most common bone resorption markers naturally secreted by osteoclasts^55^ within resorption sites. The increased expression of TRAP was characteristic for BMSC_SAM/P6_ and determined both at mRNA, as well as protein level. TRAP activity correlates not only with resorptive activity of osteoclasts, but also might be implicated in autoimmune disorders.^56^ The complex role of TRAP has been explained by its key role in both bone homeostasis and the immune system.^56^, ^57^ Many papers indicated that TRAP is not only the typical marker of osteoclasts, but also a significant player during chronic inflammation.^58^ Moreover, Solberg et al showed that *Trap* expression in osteoblasts and osteocytes could be related to the capability of this enzyme to phosphorylate the pro‐osteogenic proteins widely expressed by this cell types.^59^ Thus, the molecular significance of Trap expression in multiple cell types has not been yet well elucidated.

Additionally, in this study, the high correlation between *Trap* and lncRNA *DANCR1* has been shown. Recent data indicate the critical role of *DANCR1* in osteoclastogenesis and osteoblasts differentiation.^60^ The high expression of *DANCR1* in BMSC_SAM/P6_ might underline their similarity to osteoporotic human BMSCs that acquires osteoclast‐like phenotype. It was shown that lncDANCR is highly expressed in osteoporotic patients and promotes IL‐6 and TNF‐α expression at mRNA and protein level in human blood mononuclear cells (MNC). The lnc*DANCR1* increased resorbing activity of MNC, which can serve as a source of osteoclasts.^60^ Therefore, we believe that *DANCR1*, as a result of its involvement in osteoporosis pathology in humans, may also serve as a biomarker for osteoporosis in BMSC_SAM/P6_.

Moreover, we have established the profile of small non‐coding RNAs profile (miRNA/miR), involved in the epigenetic regulation of bone development and homeostasis. We have found that BMSC_SAM/P6_ exhibit significantly increased miR levels including miR‐7a‐5p, miR‐17‐5p, miR‐124‐3p, miR145‐3p, miR‐203pa and miR‐223‐3p. Those molecules are associated with senescence‐ and age‐dependent osteoporosis.^61^, ^62^, ^63^, ^64^, ^65^, ^66^, ^67^, ^68^ Mentioned miRNAs were highly expressed in osteoporotic BMSCs, however, in our previous articles, we have proven the dual role of the several miRNAs, including miR‐124‐3p, miR‐203a and miR‐223‐3p.^7^, ^21^ For that reason, the miRNAs biology needs to be determined. The miRNAs can be encapsulated in extracellular exosomes and/or microvesicles and released into osteoporotic tissue microenvironment. Moreover, mounting evidence show that miRNAs delivered through exosomes are present in body fluids, for example in blood, saliva and urine, thus influence distant cells of different types and may serve as diagnostic and prognostic markers.^69^, ^70^, ^71^

Finally, as a result of the accumulation of the high amount of ROS and depolarised mitochondrial membrane, BMSC_SAM/P6_ defectively expressed master regulators of osteogenesis including *Coll‐1*, *Opg* and *Opn*, leading to the reduction in extracellular matrix mineralisation. The reduced expression of *Coll‐1, Opg* and *Opn* has been previously shown in ovariectomised rat or human BMSCs derived from osteoporosis patients.^72^, ^73^, ^74^, ^75^ Our data confirmed that senile osteoporosis influence the expression of OPN, both at mRNA and protein level. Previously, OPN was described as a protective factor against postmenopausal osteoporosis development, while OPG is a well‐known inhibitor of osteoclastogenesis that protects against age‐dependent osteoporosis development. Thus, reduced expression of both *Opn* and *Opg* noted in BMSC_SAM/P6_ confirms a similar mechanism that modulates bone resorption in humans with senile osteoporosis.

Interestingly, mRNA expression for transcription factor *Runx‐2* noted in BMSC_SAM/P6_ and BMSC_BALB.c_ was comparable. However, the analysis of RUNX‐2 protein expression also confirmed disturbed osteogenic potential of BMSC_SAM/P6_. A similar tendency has been observed by Corrigan and colleges^26^ indicating the impaired osteoblast differentiation of human BMSCs derived from the osteoporotic patients. Moreover, Zannata et al showed that RUNX‐2 regulates bone formation and remodelling throughout life, and its expression profile is age‐dependent and correlates with bone mineral density (BMD).^76^

This study indicates that BMSC_SAM/P6_ exhibit a high similarity with progenitor cells isolated from osteoporotic human patients, thus becoming a novel model for in vitro study to develop new and efficient therapeutic strategies for age‐related (senile) osteoporosis. The SAM/P6 model of osteoporosis has a valuable impact on the preclinical examinations of age‐related osteoporosis and might help to develop more effective strategies of treatment. Here, we have performed profound characteristic of BMSC_SAM/P6_ cytophysiology, with particular attention on self‐renewal and multilineage potential. The BMSC_SAM/P6_ show features of ageing and senescent cells, with lowered pro‐regenerative function, related to decreased osteogenic potential and enhanced accumulation of a lipid vacuoles. Given the limited access to human cells with age‐related and senescence phenotype typical for senile osteoporosis, the BMSC_SAM/P6_ can be used successfully as a reliable model to explore and establish novel agents for osteoporosis treatment.

## CONFLICTS OF INTEREST

The authors declare no conflict of interest.

## AUTHOR CONTRIBUTION

**Mateusz Sikora:** Data curation (equal); Formal analysis (equal); Investigation (equal); Methodology (equal); Resources (equal); Software (equal); Validation (equal); Visualization (equal); Writing‐original draft (equal); Writing‐review & editing (equal). **Agnieszka Smieszek:** Conceptualization (equal); Data curation (equal); Formal analysis (equal); Investigation (equal); Methodology (equal); Resources (equal); Software (equal); Supervision (equal); Validation (equal); Visualization (equal); Writing‐original draft (equal); Writing‐review & editing (equal). **Krzysztof Marycz:** Conceptualization (equal); Funding acquisition (lead); Project administration (lead); Supervision (equal); Validation (equal); Writing‐original draft (equal); Writing‐review & editing (equal).

## Supporting information

Figure S1

## Data Availability

The data sets used in this study are available from the first author and corresponding author on reasonable request.

## References

[1] Porter JL , Varacallo M . Osteoporosis. In: *StatPearls*. StatPearls Publishing; 2020. Accessed January 2, 2021. http://www.ncbi.nlm.nih.gov/books/NBK441901/

[2] Johnston CB , Dagar M . Osteoporosis in older adults. Med Clin North Am. 2020;104(5):873‐884. 10.1016/j.mcna.2020.06.004 32773051

[3] Hernlund E , Svedbom A , Ivergård M , et al. Osteoporosis in the European Union: medical management, epidemiology and economic burden. A report prepared in collaboration with the International Osteoporosis Foundation (IOF) and the European Federation of Pharmaceutical Industry Associations (EFPIA). Arch Osteoporos. 2013;8(1‐2):136. 10.1007/s11657-013-0136-1 24113837 PMC3880487

[4] Compston J . Osteoporosis: social and economic impact. Radiol Clin North Am. 2010;48(3):477‐482. 10.1016/j.rcl.2010.02.010 20609886

[5] Föger‐Samwald U , Dovjak P , Azizi‐Semrad U , Kerschan‐Schindl K , Pietschmann P . Osteoporosis: Pathophysiology and therapeutic options. EXCLI J. 2020;19:1017‐1037. 10.17179/excli2020-2591 32788914 PMC7415937

[6] Sandhu SK , Hampson G . The pathogenesis, diagnosis, investigation and management of osteoporosis. J Clin Pathol. 2011;64(12):1042‐1050. 10.1136/jcp.2010.077842 21896577

[7] Sikora M , Marycz K , Smieszek A . Small and long non‐coding RNAs as functional regulators of bone homeostasis, acting alone or cooperatively. Molecular Therapy ‐ Nucleic Acids. 2020;21:792‐803. 10.1016/j.omtn.2020.07.017 32791451 PMC7419272

[8] Zhou C‐C , Wu Z‐P , Zou S‐J . The study of signal pathway regulating the osteogenic differentiation of bone marrow mesenchymal stem cells. Sichuan Da Xue Xue Bao Yi Xue Ban. 2020;51(6):777‐782. 10.12182/20201160103 33236600

[9] Friedenstein AJ . Determined and Inducible Osteogenic Precursor Cells. In: Ciba Foundation Symposium 11 ‐ Hard Tissue Growth, Repair and Remineralization. John Wiley and Sons. Ltd; 1973:169‐185. 10.1002/9780470719947.ch9

[10] Friedenstein AJ , Chailakhyan RK , Gerasimov UV . Bone marrow osteogenic stem cells: in vitro cultivation and transplantation in diffusion chambers. Cell Tissue Kinet. 1987;20(3):263‐272. 10.1111/j.1365-2184.1987.tb01309.x 3690622

[11] Qadir A , Liang S , Wu Z , Chen Z , Hu L , Qian A . Senile osteoporosis: the involvement of differentiation and senescence of bone marrow stromal cells. Int J Mol Sci. 2020;21(1) :349. 10.3390/ijms21010349 PMC6981793 31948061

[12] Dominici M , Le Blanc K , Mueller I , et al. Minimal criteria for defining multipotent mesenchymal stromal cells. The International Society for Cellular Therapy position statement. Cytotherapy. 2006;8(4):315‐317. 10.1080/14653240600855905 16923606

[13] Huo S‐C , Yue B . Approaches to promoting bone marrow mesenchymal stem cell osteogenesis on orthopedic implant surface. World J Stem Cells. 2020;12(7):545‐561. 10.4252/wjsc.v12.i7.545 32843913 PMC7415248

[14] Yu L , Wu Y , Liu J , et al. 3D culture of bone marrow‐derived mesenchymal stem cells (BMSCs) could improve bone regeneration in 3D‐printed porous Ti6Al4V scaffolds. Stem Cells Int. 2018;2018:1‐13. 10.1155/2018/2074021 PMC6145055 30254680

[15] Jin Y‐Z , Lee JH . Mesenchymal stem cell therapy for bone regeneration. Clin Orthop Surg. 2018;10(3):271‐278. 10.4055/cios.2018.10.3.271 30174801 PMC6107811

[16] Matsushita Y , Nagata M , Kozloff KM , et al. A Wnt‐mediated transformation of the bone marrow stromal cell identity orchestrates skeletal regeneration. Nat Commun. 2020;11(1):332. 10.1038/s41467-019-14029-w 31949165 PMC6965122

[17] Frank O , Heim M , Jakob M , et al. Real‐time quantitative RT‐PCR analysis of human bone marrow stromal cells during osteogenic differentiation in vitro. J Cell Biochem. 2002;85(4):737‐746. 10.1002/jcb.10174 11968014

[18] Yu H , Cheng J , Shi W , et al. Bone marrow mesenchymal stem cell‐derived exosomes promote tendon regeneration by facilitating the proliferation and migration of endogenous tendon stem/progenitor cells. Acta Biomater. 2020;106:328‐341. 10.1016/j.actbio.2020.01.051 32027991

[19] Zhang Y , Liu Y , Liu H , Tang WH . Exosomes: biogenesis, biologic function and clinical potential. Cell Biosci. 2019;9: 10.1186/s13578-019-0282-2 PMC6377728 30815248

[20] Draebing T , Heigwer J , Juergensen L , Katus HA , Hassel D . Extracellular vesicle‐delivered bone morphogenetic proteins: a novel paracrine mechanism during embryonic development. bioRxiv. 2018. 10.1101/321356

[21] Smieszek A , Marcinkowska K , Pielok A , Sikora M , Valihrach L , Marycz K . The Role of miR‐21 in osteoblasts‐osteoclasts coupling in vitro. Cells. 2020;9(2):479. 10.3390/cells9020479 PMC7072787 32093031

[22] Luo Z , Lin J , Sun Y , Wang C , Chen J . Bone marrow stromal cell‐derived exosomes promote muscle healing following contusion through macrophage polarization. Stem Cells Dev. 2021;30(3):135‐148. 10.1089/scd.2020.0167 33323007

[23] Huang T , Yu Z , Yu Q , et al. Inhibition of osteogenic and adipogenic potential in bone marrow‐derived mesenchymal stem cells under osteoporosis. Biochem Biophys Res Comm. 2020;525(4):902‐908. 10.1016/j.bbrc.2020.03.035 32171528

[24] Huang Y , Yin Y , Gu Y , et al. Characterization and immunogenicity of bone marrow‐derived mesenchymal stem cells under osteoporotic conditions. Sci China Life Sci. 2020;63(3):429‐442. 10.1007/s11427-019-1555-9 31879847

[25] Coipeau P , Rosset P , Langonné A , et al. Impaired differentiation potential of human trabecular bone mesenchymal stromal cells from elderly patients. Cytotherapy. 2009;11(5):584‐594. 10.1080/14653240903079385 19626496

[26] Corrigan MA , Coyle S , Eichholz KF , Riffault M , Lenehan B , Hoey DA . Aged osteoporotic bone marrow stromal cells demonstrate defective recruitment, mechanosensitivity, and matrix deposition. CTO. 2019;207(2):83‐96. 10.1159/000503444 31655814

[27] Śmieszek A , Czyrek A , Basinska K , et al. effect of metformin on viability, morphology, and ultrastructure of mouse bone marrow‐derived multipotent mesenchymal stromal cells and Balb/3T3 embryonic fibroblast cell line. Biomed Res Int. 2015;2015:1‐14. 10.1155/2015/769402 PMC4430655 26064951

[28] Soleimani M , Nadri S . A protocol for isolation and culture of mesenchymal stem cells from mouse bone marrow. Nat Protoc. 2009;4(1):102‐106. 10.1038/nprot.2008.221 19131962

[29] Śmieszek A , Tomaszewski K , Kornicka‐Garbowska K , Marycz K . Metformin promotes osteogenic differentiation of adipose‐derived stromal cells and exerts pro‐osteogenic effect stimulating bone regeneration. Journal of Clinical Medicine. 2018;7:482. 10.3390/jcm7120482 PMC6306720 30486321

[30] Śmieszek A , Stręk Z , Kornicka K , Grzesiak J , Weiss C , Marycz K . Antioxidant and anti‐senescence effect of metformin on mouse olfactory ensheathing cells (mOECs) may be associated with increased brain‐derived neurotrophic factor levels—an ex vivo study. Int J Mol Sci. 2017;18(4):872. 10.3390/ijms18040872 PMC5412453 28425952

[31] Marycz K , Weiss C , Śmieszek A , Kornicka K . Evaluation of oxidative stress and mitophagy during adipogenic differentiation of adipose‐derived stem cells isolated from equine metabolic syndrome (EMS) horses. Stem Cells Int. 2018;2018:1–18. 10.1155/2018/5340756 PMC6011082 29977307

[32] Targonska S , Sikora M , Marycz K , Smieszek A , Wiglusz RJ . Theranostic Applications of Nanostructured Silicate‐Substituted Hydroxyapatite Codoped with Eu3+ and Bi3+ Ions—A Novel Strategy for Bone Regeneration. ACS Biomater Sci Eng. Published online September 22, 2020. 10.1021/acsbiomaterials.0c00824 33449662

[33] Seweryn A , Pielok A , Lawniczak‐Jablonska K , et al. Zirconium oxide thin films obtained by atomic layer deposition technology abolish the anti‐osteogenic effect resulting from miR‐21 inhibition in the pre‐osteoblastic MC3T3 cell line. Int J Nanomedicine. 2020;15:1595‐1610. 10.2147/IJN.S237898 32210554 PMC7069564

[34] Smieszek A , Seweryn A , Marcinkowska K , et al. Titanium dioxide thin films obtained by atomic layer deposition promotes osteoblasts’ viability and differentiation potential while inhibiting osteoclast activity—potential application for osteoporotic bone regeneration. Materials. 2020;13(21):4817. 10.3390/ma13214817 PMC7662580 33126628

[35] Sikora M , Marcinkowska K , Marycz K , Wiglusz RJ , Śmieszek A . The potential selective cytotoxicity of poly (L‐ lactic acid)‐based scaffolds functionalized with Nanohydroxyapatite and Europium (III) ions toward osteosarcoma cells. Materials (Basel). 2019;12(22): 10.3390/ma12223779 PMC6888250 31752084

[36] Bindokas VP , Jordán J , Lee CC , Miller RJ . Superoxide production in rat hippocampal neurons: selective imaging with hydroethidine. J Neurosci. 1996;16(4):1324‐1336. 8778284 10.1523/JNEUROSCI.16-04-01324.1996 PMC6578569

[37] Vu TH , Shipley JMichael , Bergers G , et al. MMP‐9/Gelatinase B Is a key regulator of growth plate angiogenesis and apoptosis of hypertrophic chondrocytes. Cell. 1998;93(3):411‐422. 9590175 10.1016/s0092-8674(00)81169-1 PMC2839071

[38] Kadiroğlu ET , Akbalık ME , Karaöz E , et al. Calvarial bone defects in ovariectomised rats treated with mesenchymal stem cells and demineralised freeze‐dried bone allografts. Folia Morphologica. 2020;79(4):720‐735. 10.5603/FM.a2020.0001 31930468

[39] Yousefzadeh N , Kashfi K , Jeddi S , Ghasemi A . Ovariectomized rat model of osteoporosis: a practical guide. EXCLI J. 2020;19:89‐107. 10.17179/excli2019-1990 32038119 PMC7003643

[40] Petrenko Y , Vackova I , Kekulova K , et al. A Comparative analysis of multipotent mesenchymal stromal cells derived from different sources, with a focus on neuroregenerative potential. Sci Rep. 2020;10(1):4290. 10.1038/s41598-020-61167-z 32152403 PMC7062771

[41] Infante A , Rodríguez CI . Osteogenesis and aging: lessons from mesenchymal stem cells. Stem Cell Res Ther. 2018;9(1):244. 10.1186/s13287-018-0995-x 30257716 PMC6158877

[42] Alicka M , Kornicka‐Garbowska K , Kucharczyk K , Kępska M , Rӧcken M , Marycz K . Age‐dependent impairment of adipose‐derived stem cells isolated from horses. Stem Cell Res Ther. 2020;11(1):4. 10.1186/s13287-019-1512-6 31900232 PMC6942290

[43] Kornicka K , Marycz K , Tomaszewski KA , Marędziak M , Śmieszek A . The effect of age on osteogenic and adipogenic differentiation potential of human adipose derived stromal stem cells (hASCs) and the impact of stress factors in the course of the differentiation process. Oxid Med Cell Longev. 2015;2015:1‐20. 10.1155/2015/309169 PMC4515302 26246868

[44] Chen Y , Wang W , Liu F , Tang L , Tang R , Li W . Apoptotic effect of mtrix metalloproteinases 9 in the development of diabetic retinopathy. Int J Clin Exp Pathol. 2015;8(9):10452‐10459. 26617754 PMC4637569

[45] Abdollahi M , Larijani B , Rahimi R , Salari P . Role of oxidative stress in osteoporosis. Therapy. 2005;2(5):787‐796. 10.2217/14750708.2.5.787

[46] Domazetovic V , Marcucci G , Iantomasi T , Brandi ML , Vincenzini MT . Oxidative stress in bone remodeling: role of antioxidants. Clin Cases Miner Bone Metab. 2017;14(2):209‐216. 10.11138/ccmbm/2017.14.1.209 29263736 PMC5726212

[47] Tait SWG , Green DR . Caspase‐independent cell death: leaving the set without the final cut. Oncogene. 2008;27(50):6452‐6461. 10.1038/onc.2008.311 18955972 PMC2635930

[48] Kroemer G , Martin SJ . Caspase‐independent cell death. Nat Med. 2005;11(7):725‐730. 10.1038/nm1263 16015365

[49] Ishihara N , Eura Y , Mihara K . Mitofusin 1 and 2 play distinct roles in mitochondrial fusion reactions via GTPase activity. J Cell Sci. 2004;117(Pt 26):6535‐6546. 10.1242/jcs.01565 15572413

[50] Belov SV , Lobachevsky YP , Danilejko YK , et al. The role of mitochondria in the dual effect of low‐temperature plasma on human bone marrow stem cells: from apoptosis to activation of cell proliferation. Appl Sci. 2020;10(24):8971. 10.3390/app10248971

[51] Ren L , Chen X , Chen X , Li J , Cheng B , Xia J . Mitochondrial dynamics: fission and fusion in fate determination of mesenchymal stem cells. Front Cell Dev Biol. 2020;8. 10.3389/fcell.2020.580070 PMC7593605 33178694

[52] Bingol B , Sheng M . Mechanisms of mitophagy: PINK1, Parkin, USP30 and beyond. Free Radic Biol Med. 2016;100:210‐222. 10.1016/j.freeradbiomed.2016.04.015 27094585

[53] Matsuda S , Kitagishi Y , Kobayashi M . Function and characteristics of PINK1 in mitochondria. Oxid Med Cell Longev. 2013;2013:1‐6. 10.1155/2013/601587 PMC3600171 23533695

[54] Feng X , Yin W , Wang J , Feng L , Kang YJ . Mitophagy promotes the stemness of bone marrow‐derived mesenchymal stem cells. Exp Biol Med (Maywood). 2021;246(1):97‐105. 10.1177/1535370220964394 33172301 PMC7797993

[55] Kuo T‐R , Chen C‐H . Bone biomarker for the clinical assessment of osteoporosis: recent developments and future perspectives. Biomark Res. 2017;5. 10.1186/s40364-017-0097-4 PMC5436437 28529755

[56] Hayman AR . Tartrate‐resistant acid phosphatase (TRAP) and the osteoclast/immune cell dichotomy. Autoimmunity. 2008;41(3):218‐223. 10.1080/08916930701694667 18365835

[57] Ginaldi L , Di Benedetto MC , De Martinis M . Osteoporosis, inflammation and ageing. Immun Ageing. 2005;2:14. 10.1186/1742-4933-2-14 16271143 PMC1308846

[58] Park JK , Rosen A , Saffitz JE , et al. Expression of cathepsin K and tartrate‐resistant acid phosphatase is not confined to osteoclasts but is a general feature of multinucleated giant cells: systematic analysis. Rheumatology. 2013;52(8):1529‐1533. 10.1093/rheumatology/ket184 23674817

[59] Solberg LB , Brorson S‐H , Stordalen GA , Bækkevold ES , Andersson G , Reinholt FP . Increased tartrate‐resistant acid phosphatase expression in osteoblasts and osteocytes in experimental osteoporosis in rats. Calcif Tissue Int. 2014;94(5):510‐521. 10.1007/s00223-013-9834-3 24395179 PMC4148331

[60] Tong X , Gu P , Xu S , Lin X . Long non‐coding RNA‐DANCR in human circulating monocytes: a potential biomarker associated with postmenopausal osteoporosis. Biosci Biotechnol Biochem. 2015;79(5):732‐737. 10.1080/09168451.2014.998617 25660720

[61] Zhou M , Ma J , Chen S , Chen X , Yu X . MicroRNA‐17‐92 cluster regulates osteoblast proliferation and differentiation. Endocrine. 2014;45(2):302‐310. 10.1007/s12020-013-9986-y 23673870

[62] Tang L , Yin Y , Liu J , Li Z , Lu X . MiR‐124 attenuates osteoclastogenic differentiation of bone marrow monocytes via targeting Rab27a. Cell Physiol Biochem. 2017;43(4):1663‐1672. 10.1159/000484027 29045940

[63] Tang J , Lin X , Zhong J , et al. miR‐124 regulates the osteogenic differentiation of bone marrow‐derived mesenchymal stem cells by targeting Sp7. Mol Med Rep. 2019;49. 10.3892/mmr.2019.10054 30896834

[64] Hao W , Liu H , Zhou L , et al. MiR‐145 regulates osteogenic differentiation of human adipose‐derived mesenchymal stem cells through targeting FoxO1. Exp Biol Med (Maywood). 2018;243(4):386‐393. 10.1177/1535370217746611 29249185 PMC6022928

[65] Jin Y , Hong F , Bao Q , et al. MicroRNA‐145 suppresses osteogenic differentiation of human jaw bone marrow mesenchymal stem cells partially via targeting semaphorin 3A. Connect Tissue Res. 2020;61(6):577‐585. 10.1080/03008207.2019.1643334 31305177

[66] Tang Y , Zheng L , Zhou J , et al. MiR‐203‐3p participates in the suppression of diabetes‐associated osteogenesis in the jaw bone through targeting Smad. Int J Mol Med. 2018;41. 10.3892/ijmm.2018.3373 PMC5819914 29328402

[67] Zhang S , Liu YI , Zheng Z , et al. MicroRNA‐223 suppresses osteoblast differentiation by inhibiting DHRS3. Cell Physiol Biochem. 2018;47(2):667‐679. 10.1159/000490021 29794437

[68] Li X , Zheng Y , Zheng Y , et al. Circular RNA CDR1as regulates osteoblastic differentiation of periodontal ligament stem cells via the miR‐7/GDF5/SMAD and p38 MAPK signaling pathway. Stem Cell Res Ther. 2018;9(1):232. 10.1186/s13287-018-0976-0 30170617 PMC6119336

[69] Cheng L , Sun X , Scicluna BJ , Coleman BM , Hill AF . Characterization and deep sequencing analysis of exosomal and non‐exosomal miRNA in human urine. Kidney Int. 2014;86(2):433‐444. 10.1038/ki.2013.502 24352158

[70] Gallo A , Tandon M , Alevizos I , Illei GG . The majority of microRNAs detectable in serum and saliva is concentrated in exosomes. PLoS One. 2012;7(3):e30679. 10.1371/journal.pone.0030679 22427800 PMC3302865

[71] Zhao F , Cheng LI , Shao Q , et al. Characterization of serum small extracellular vesicles and their small RNA contents across humans, rats, and mice. Sci Rep. 2020;10(1):4197. 10.1038/s41598-020-61098-9 32144372 PMC7060188

[72] Ma Z‐P , Zhang Z‐F , Yang Y‐F , Yang Y . Sesamin promotes osteoblastic differentiation and protects rats from osteoporosis. Med Sci Monit. 2019;25:5312‐5320. 10.12659/MSM.915529 31314750 PMC6659468

[73] Liu X , Bao C , Xu HHK , et al. Osteoprotegerin gene‐modified BMSCs with hydroxyapatite scaffold for treating critical‐sized mandibular defects in ovariectomized osteoporotic rats. Acta Biomater. 2016;42:378‐388. 10.1016/j.actbio.2016.06.019 27318268

[74] Meng B , Wu D , Cheng Y , et al. Interleukin‐20 differentially regulates bone mesenchymal stem cell activities in RANKL‐induced osteoclastogenesis through the OPG/RANKL/RANK axis and the NF‐κB, MAPK and AKT signalling pathways. Scand J Immunol. 2020;91(5):e12874. 10.1111/sji.12874 32090353

[75] He W , Chen L , Huang Y , et al. Synergistic effects of recombinant Lentiviral‐mediated BMP2 and TGF‐beta3 on the osteogenic differentiation of rat bone marrow mesenchymal stem cells in vitro. Cytokine. 2019;120:1‐8. 10.1016/j.cyto.2019.03.020 30991228

[76] Zanatta M , Valenti MT , Donatelli L , Zucal C , Dalle CL . Runx‐2 gene expression is associated with age‐related changes of bone mineral density in the healthy young‐adult population. J Bone Miner Metab. 2012;30(6):706‐714. 10.1007/s00774-012-0373-1 22903460

