# Supplementary figures and images for: Bone marrow stromal cells (BMSCs CD45‐/CD44+/CD73+/CD90+) isolated from osteoporotic mice SAM/P6 as a novel model for osteoporosis investigation
Source: J Cell Mol Med. 2021 Jun 1;25(14):6634–51. doi: 10.1111/jcmm.16667 (PMC8278098; doi:10.1111/jcmm.16667)

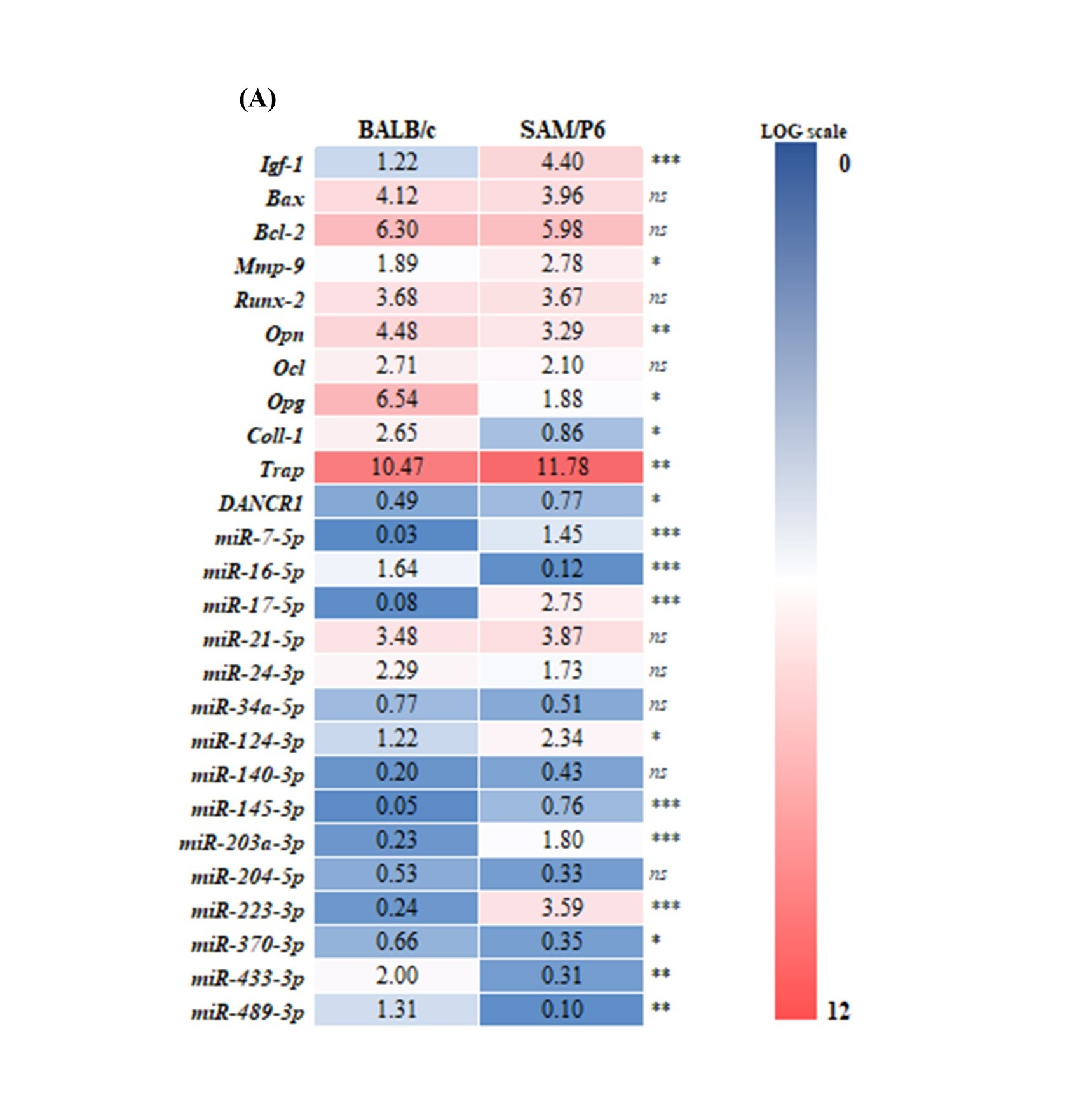

Supplement: Supplementary file 1 — Figure S1 [file JCMM-25-6634-s001.jpg]
